# Strong Host Modulation of Rhizosphere‐to‐Endosphere Microbial Colonisation in Natural Populations of the Pan‐Palaeotropical Keystone Grass Species, *Themeda triandra*

**DOI:** 10.1002/ece3.71595

**Published:** 2025-06-18

**Authors:** Riley J. Hodgson, Christian Cando‐Dumancela, Craig Liddicoat, Sunita A. Ramesh, Robert A. Edwards, Martin F. Breed

**Affiliations:** ^1^ College of Science and Engineering Flinders University Bedford Park South Australia Australia

**Keywords:** endosphere, microbial ecology, neutral theory model, rhizosphere, *Themeda triandra*, two‐step selection process

## Abstract

Soil microbiota can colonise plant roots through a two‐step selection process, involving recruitment of microbiota first from bulk soil into plant rhizospheres, then into root endospheres. This process is poorly understood in all but a few model species (e.g., *Arabidopsis*), which is surprising given its fundamental role in plant and soil ecology. Here, we examined the microbial community assembly processes across the rhizospheres and root endospheres in eight natural populations of the pan‐palaeotropical C4 grass, *Themeda triandra* , in southern Australia. Using a space‐for‐time substitution approach, we assessed whether bacterial root colonisation patterns conformed to the two‐step model and tested whether community assembly was driven more by deterministic or stochastic processes. Our results show that the two‐step selection process shaped bacterial recruitment dynamics across these natural *T. triandra* populations, and we provide clear evidence that host plants influence microbial assembly via deterministic pressures that produce strong community convergence within endospheres. These findings highlight the central role of host filtering in shaping a conserved ‘core’ endosphere microbiome. However, limited understanding of these endosphere communities constrains efforts to harness these important relationships to, for example, improve plant propagation and revegetation practices.

## Graphical abstract

This study investigates microbial assembly in rhizospheres and root endospheres of the C4 grass *Themeda triandra* across eight natural populations in southern Australia. Results show that root colonisation follows a two‐step selection process, shaped by deterministic factors including host plant influence and environmental conditions. These findings enhance our understanding of plant‐microbe interactions and their ecological significance in non‐model species.

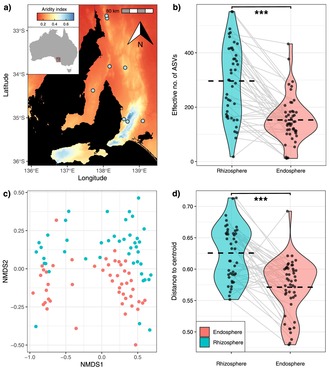

## 1 Introduction

Soil microbiota have important roles in ecosystem functioning as they help to drive ecological processes (e.g., nutrient cycling) and are important contributors to plant growth and fitness (David et al. 2019; Wang et al. 2019; Choi et al. 2021). These soil microbiota commonly interact with plants via plant–soil feedbacks, where plants release organic exudates into the soil via their roots which then influence microbial community structure and diversity patterns (Bever et al. 2010). In turn, microbiota can provide their host plants with essential nutrients, protection against pathogens, and growth or fitness advantages via the release of metabolites and/or hormones (de Vries et al. 2020; Thiergart et al. 2020; Yang et al. 2021). Though generally poorly understood in non‐model systems, a better understanding of these plant–soil feedbacks has promise to help ecosystem managers make more informed decisions about how to reintroduce or promote plant species (Breed et al. 2019; de Vries et al. 2020; Thiergart et al. 2020), especially during plant propagation, translocation and revegetation efforts (Peixoto et al. 2022; Robinson et al. 2023).

Soil microbiota can colonise plant roots via a two‐step selection process, where certain soil microbiota are selectively recruited from bulk soil into plant rhizospheres (the soil and associated microbiota surrounding roots), and then into the root endosphere (the microbiota inside roots) via plant‐mediated processes (Lundberg et al. 2012; Bulgarelli et al. 2013; Urbina et al. 2018). This two‐step selection process is promoted by the deposition of cells and organic exudates that attract microbiota into rhizospheres from bulk soils. From the rhizosphere, microbiota can enter into plant roots to form root endospheres via regulation of the plant's immune system (Bulgarelli et al. 2013). The differentiation in beneficial microbiota observed across plant rhizospheres and endospheres can be linked to how microbiota are selected by their host plants (Urbina et al. 2018; Stopnisek and Shade 2021). However, the assembly dynamics responsible for the microbial composition of rhizospheres and root endospheres are poorly explored, especially in non‐model organisms (e.g., unlike for *Arabidopsis thaliana* ) (Naylor and Coleman‐Derr 2018; Sasse et al. 2018; Thiergart et al. 2020). Indeed, in natural systems, we might expect assembly processes to depend strongly on local environmental conditions, such as site‐level aridity, but these dynamics remain poorly understood (Petipas et al. 2017; Hodgson et al. 2024).

Rhizospheres and endospheres can affect host plant fitness (Zhang et al. 2020; Durán et al. 2022; Ling et al. 2022). Indeed, it is not only the most abundant microbial taxa that are important for plants; rare microbial taxa can also promote plant health and affect microbial community dynamics (Jousset et al. 2017; Neu et al. 2021; Custer et al. 2023). Identifying rare and abundant taxa, and taxa whose abundances are variable across ecological contexts (i.e., conditionally rare and/or abundant), can provide insight into rhizosphere and endosphere recruitment (Logares et al. 2014; Xue et al. 2018; Zhang et al. 2018). By separately considering these components of microbial communities, we can determine potential differences in their recruitment (i.e., how important are rare vs. abundant taxa?). Highly diverse recruitment strategies can highlight the importance of microbiota fulfilling multiple functions for their hosts, by for example, offering long‐term protection against stress or disturbance through generating functional redundancy (Naeem et al. 1994; Louca et al. 2018). Therefore, characterising the structure of microbial communities—and the microbial taxa that are selected for by host plants—can identify functionally important microbial taxa, plus the recruitment strategies used by the host plants (Hamonts et al. 2018; Risely 2020; Ling et al. 2022).

Neutral ecological theory has previously been used to describe the assembly of microbial communities in terms of deterministic versus neutral processes (Ofiţeru et al. 2010; Stopnisek and Shade 2021). While neutral models are built on assumptions of functional equivalence among taxa (Zhou and Ning 2017; Rocha 2018), they enable direct comparisons of microbial communities based on whether taxa follow patterns expected under neutral processes, which operate stochastically through random birth, death, and dispersal (Burns et al. 2016; Stopnisek and Shade 2021). Indeed, taxa deviating from these models may be affected more by environment/host selection. Different types of deterministic selection processes can also be inferred by predicting rates of phylogenetic or taxonomic community turnover among communities, which would be expected under random population fluctuations (i.e., ecological drift) (Stegen et al. 2013). These include heterogeneous selection (environments lead to greater phylogenetic turnover) or homogeneous selection (environments reduce phylogenetic turnover) (Stegen et al. 2012, 2013; Ning et al. 2020). Furthermore, taxonomic turnover can also be used to predict dispersal rates via processes like homogenising dispersal (communities become more similar than expected due to high movement) or dispersal limitation (drift causes greater differentiation in communities). Overall, these methods offer a useful option to understand the complex processes shaping different microbiota.

*Themeda triandra* is a pan‐palaeotropical C4 grass species that is globally dominant in many grassland ecosystems (Snyman et al. 2013). While this plant is widely distributed, grasslands are in global decline (Murphy et al. 2016; Bardgett et al. 2021), and there is a need to build new knowledge that assists the recovery of grassland ecosystems that are resilient to climate change (Gopal and Gupta 2016; Brinkman et al. 2017; Larson et al. 2022). Soil microbiota are known to strongly associate with *T. triandra* (Hodgson et al. 2024), and can aid the growth and fitness of this plant (Hassen and Labuschagne 2010; Petipas et al. 2017). Microbial communities linked to *T. triandra* fitness may also be susceptible to climate change impacts, including warming temperatures, increased CO_2_ and desertification (Hayden et al. 2012; Tang et al. 2021). Therefore, further understanding the composition of the microbial communities that directly interact with *T. triandra* root structures—such as those surrounding (i.e., rhizospheres) and within (i.e., endospheres) roots—across a diversity of climatic and soil conditions is a key step for identifying the microbial taxa and environmental circumstances that should promote the growth and fitness of this plant (Hayden et al. 2012; Snyman et al. 2013; Gonzalez et al. 2018).

Here, we examined the two‐step selection process of *T. triandra* through a microbial community assembly lens, focusing on regional variation in its rhizospheres and endospheres. This study builds on our previous research by Hodgson et al. (2024), which investigated the first step of the selection process—the movement of microbiota from bulk soil into rhizospheres using a space‐for‐time substitution—and found that rhizospheres exhibited lower diversity and distinct bacterial communities compared to surrounding soils. While we do not examine bulk soils in this study, we aim to address whether the bacterial communities within the endospheres of *T. triandra* originate from the diversity found in rhizospheres and whether regional differences affect patterns of endosphere colonisation by rhizosphere microbiota. Using 16S rRNA amplicon sequencing, we characterised the bacterial communities within rhizospheres and root endospheres of eight naturally occurring *T. triandra* populations along a southern Australian aridity gradient. Our focus is on the changing patterns between the rhizosphere and endosphere bacterial communities, the second step of the two‐step selection process. To further investigate these dynamics, we used neutral theory models and diversity‐based analyses to explore the different processes driving selection and bacterial colonisation across these different ecological populations. We posed the following research questions: (1) Do *T. triandra* rhizosphere and root endosphere bacterial communities align with the processes described in the two‐step selection process, with reduced bacterial diversity in the endosphere compared to the rhizosphere? (2) Is there evidence of different deterministic or stochastic assembly processes within each site influencing the assembly of rhizosphere and endosphere bacterial communities? (3) Are the bacterial communities in the *T. triandra* endosphere within each site entirely constrained by the diversity of bacteria available in the rhizosphere, or are there other sources of bacterial recruitment?

## 2 Methods

### 2.1 Study Species

*Themeda triandra* (Forssk.) is a pan‐palaeotropical, perennial C4 grass that forms tussocks and generally reaches heights of 1 m, often dominating other species (Snyman et al. 2013; Dunning et al. 2017). As a keystone species, *T. triandra* is important for supporting invertebrate communities across stable environments (Snyman et al. 2013), and it has important associations with fire—for instance, it deposits flammable leaf litter that accumulates during growth, and its seeds respond well to smoke and high temperatures (Baxter et al. 1994; Ghebrehiwot et al. 2012). The seeds of *T. triandra* germinate best after long dormancy periods with substantial variation across regions, making it a difficult species to cultivate (Saleem et al. 2009; Farley et al. 2013; Hancock and Hughes 2014).

### 2.2 Observational Field Experiment

**FIGURE 1:**
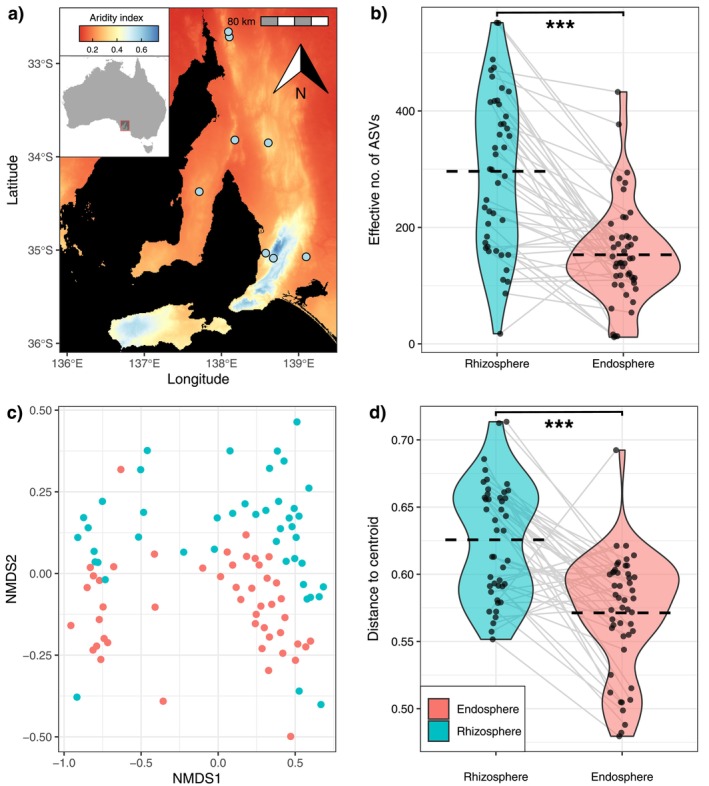
(a) Map showing Australia and the sampling locations of *Themeda triandra* populations (blue points) across a strong aridity gradient in southern Australia. (b) Bacterial alpha diversity as effective number of ASVs in *T. triandra* rhizospheres. (c) NMDS ordination showing the differences in bacterial community composition between rhizospheres (blue) and endospheres (red). (d) Distance to centroid of samples comparing rhizosphere (blue) and endosphere (red) samples, calculated from Bray–Curtis dissimilarity.

In December 2021, soil and plant tissue samples were collected from six *T. triandra* individuals across eight sites along an aridity gradient in southern Australia (aridity index values 0.318–0.903), as described in Hodgson et al. (2024) (Table 1; Figure 1a). There was no correlation between pairwise geographic distances and aridity differences between sites (Mantel: *p* = 0.489; *r* = −0.021). Mean annual aridity index data (annual precipitation/annual potential evaporation) were obtained from the Atlas of Living Australia (Belbin 2011; ALA 2014) spatial portal, using the aridity index layer (UNEP 1992; Middleton and Thomas 1997).

**TABLE 1:** *Themeda triandra* sampling sites across southern Australia.

Site name	Latitude, longitude	Aridity index	Sampling date
Alligator Gorge	−32.71487, 138.10172	0.445	15 December 2021
Barlunga Gap	−33.82000, 138.17392	0.347	14 December 2021
Frahn's Farm	−35.07231, 139.09781	0.454	19 December 2021
Maitland	−34.37366, 137.71203	0.453	21 December 2021
Mount Maria	−32.65862, 138.08985	0.318	16 December 2021
Neagles Rock Reserve	−33.85031, 138.60674	0.651	14 December 2021
Scott Creek	−35.08720, 138.67266	0.903	19 December 2021
Sturt Gorge	−35.03311, 138.57324	0.634	13 December 2021

The six *T. triandra* plants were sampled from within a 25 × 25 m area at each study site using coordinates from a random number table; for each coordinate, the nearest plant was sampled. We profiled the diversity and community composition of bacteria in the rhizospheres and root endospheres of these plants using 16S rRNA amplicon sequencing (described below).

### 2.3 Isolation and Extraction of Rhizosphere and Endosphere DNA

Microbial DNA from the rhizospheres was obtained following the protocol outlined in McPherson et al. (2018) and detailed in Hodgson et al. (2024). Briefly, sampled roots were washed in 0.02% Silwet L‐77 amended PBS buffer and vortexed, before being filtered at 100 μm and centrifuged, prior to DNA extraction. *T. triandra* endospheres were extracted by removing as many bacteria and DNA as possible from root surfaces and subsequently extracting the DNA directly from these ‘cleaned’ root tissues. To determine the best methods of isolating *T. triandra* endosphere DNA, we ran a pilot study to compare methods of root cleaning via washing, bleaching and sonicating root surfaces (see Figures S1–S3). Based on our pilot study, roots were sonicated on ice in 0.02% Silwet L‐77 amended PBS buffer at 30% amplitude for five 30 s alternating burst and rest periods over 5 min. Following this, roots underwent a series of five washes in this sterilised amended PBS buffer solution. Root endosphere samples were pulverised with metal beads for 1 min using bead beating solution (PowerSoil Kit, Qiagen, Hilden, Germany). We then extracted DNA from both rhizosphere and endosphere samples using the DNeasy PowerLyzer PowerSoil Kit (Qiagen, Hilden, Germany) following the manufacturer's protocols.

### 2.4 Amplification, Sequencing and Bioinformatics

Amplicon libraries of the 16S rRNA V3‐4 gene region were developed by the Australian Genome Research Facility (AGRF, Melbourne, Australia). Samples were PCR amplified with the forward primer, 341F (CCTAYGGGRBGCASCAG), and reverse primer, 806R (GGACTACNNGGGTATCTAAT). DNA libraries were sequenced on an Illumina MiSeq platform with 300 base pair paired‐end sequences. We used the DADA2 bioinformatic pipeline through QIIME2 (Bolyen et al. 2019) to infer identity profiles from amplicon sequence data by matching against the SILVA database (Version 138.1) (Wang et al. 2007; Quast et al. 2013) using a naïve Bayesian classifier (Wang et al. 2007; Callahan et al. 2016). We produced a table of unique amplicon sequence variants (ASVs), including their abundances and taxonomic annotations. All ASVs that were not assigned as bacteria and associated with mitochondria or chloroplasts were removed. We also removed taxa that did not occur in at least two samples to avoid non‐representative taxa.

### 2.5 Statistics

All analyses were done in R version 4.0.2 (R Core Team 2022). We rarefied samples to 11,491 reads without replacement to normalise for variation in library size across samples and to maintain a constant sampling effort for downstream analyses at both within and across sites (Figure S4). We estimated alpha diversity as effective number of ASVs (eff. no. ASVs.), which were calculated as the exponential transformation of Shannon's diversity index (Jost 2006), and Faith's phylogenetic diversity (Kembel et al. 2010). We compared these values across rhizospheres and endospheres using linear mixed‐effects models (LMM) with the *lmer* function in the lme4 package in R (Bates et al. 2015). Plant compartment (i.e., endosphere, rhizosphere) was treated as a fixed effect, and plant ID was included as a random effect to account for resampling the same individuals across rhizospheres and endospheres. Model significance was tested using a Wald chi‐squared test, and pairwise tests were made using multiple comparisons with Tukey contrasts through the *glht* function of the multcomp package (Hothorn et al. 2008). We also assessed the number of unique taxa within each belowground compartment and across sites using the Microeco package in R (Liu et al. 2020).

We visualised bacterial communities using non‐metric multidimensional scaling ordination (NMDS) with Bray–Curtis distances. We also performed NMDS ordinations using β mean nearest taxon distance (βMNTD), and weighted unifrac (wunifrac) distances, to account for phylogenetic influences (Kembel et al. 2010). Compositional differences between bacterial community compartments in the endospheres and rhizospheres samples were tested via permutational multivariate analysis of variance (PERMANOVA) using the *adonis2* function in vegan (Oksanen et al. 2019). Here, plant ID was included as a strata variable to account for repeated sampling of individual plants across both the rhizospheres and endospheres. When testing the effect of aridity on community composition, we also used compartment (rhizosphere or endosphere) as a strata variable to account for variation between these two community types. We also assessed for homogeneity of group dispersion with vegan's *betadisper* function (Oksanen et al. 2019). Visualisations of the relative abundance of the top 11 phyla across treatments were performed using the *plot_bar* function in Phyloseq (McMurdie and Holmes 2013). Rare phyla that made up < 0.5% of the total relative abundance were grouped as ‘Other minor phyla’.

#### 2.5.1 Neutral Theory Models

We compared bacterial ASVs found in rhizospheres and endospheres to a neutral model of microbial community assembly to assess host plant selection processes on these taxa (Sloan et al. 2007; Burns et al. 2016; Stopnisek and Shade 2021). This was done by comparing taxa across sites, or indeed, within root compartments to the Sloan neutral model, which assumes that community structures are principally driven by stochastic processes (i.e., reproduction, mortality, speciation, births, deaths) (Sloan et al. 2007). Although these models can underrepresent taxa or species that are deterministically selected (Stopnisek and Shade 2021), we are still able to use them to hypothesise functionally useful ASVs that may play key roles in *T. triandra* microbiomes. ASVs outside the upper confidence intervals of the neutral model were inferred as those to have undergone positive selection, while those below the lower confidence interval were assumed to be under negative selection pressure.

We also tested the contribution of different community assembly processes on shaping microbiota across the rhizospheres and endospheres within and across each sampling site. Using the R package iCAMP (Ning et al. 2020), we first assessed pairwise sample differences using a null model of the β nearest taxon index (βNTI). A βNTI < −2 indicates homogeneous selection, while a βNTI > 2 suggests heterogeneous selection. Sample comparisons yielding βNTI values between −2 and 2 were considered not to be under any significant influence of selection (Stegen et al. 2013; Ning et al. 2020). These comparisons were further evaluated using Raup–Crick values based on Bray–Curtis dissimilarity (RCbray). RCbray values > 0.95 indicated the presence of dispersal limitation alongside heightened influence of drift, while values < −0.95 suggest homogenising dispersal. RCbray values between −0.95 and 0.95 indicate that drift was operating alone. To compare the influence of different community assembly processes in rhizosphere and endosphere communities, we calculated the percentage of connections associated with each process in each sampling site: homogeneous selection, heterogeneous selection, homogenising dispersal, dispersal limitation acting with drift, and drift acting alone.

#### 2.5.2 Conditional Abundances of Bacterial ASVs

To examine the rarity of bacteria across our dataset, we assigned ASVs to three groups—abundant, moderate and rare—according to whether they met relative abundance thresholds according to Xue et al. (2018). ASVs with ≥ 1% relative abundance in any sample within sampling sites were classified as abundant taxa (AT), while ASVs with < 0.01% relative abundance were considered rare taxa (RT). ASVs between these values (i.e., ≥ 0.01 but < 1%) were considered moderate taxa (MT). Bacterial ASVs that were of relative abundances of ≥ 0.01% in all sites, but ≥ 1% in at least one site, were considered conditionally abundant taxa (CAT), whereas those that were found to be of < 1% relative abundance in all sites, but < 0.01% in at least one site, were conditionally rare taxa (CRT). The ASVs that had instances where relative abundance was at least < 0.01% in one site, but ≥ 1% in another, were considered conditionally rare and abundant taxa (CRAT). The community composition of ASVs in these categories was visualised at the phylum level with chord diagrams using the R package circlize (Gu et al. 2014).

The different abundance categories were then compared against neutral models (described above) to investigate whether these ASVs underwent selection by the host plants. ASVs which were MT, RT, and CRT were examined separately, whereas ASVs that were CRAT and CAT were combined due to the low numbers of taxa in these groupings.

#### 2.5.3 Differential Abundance

We determined differentially abundance of taxa across compartments and sites using the R package ANCOMBC (Lin and Peddada 2020) with function *ancombc2* to reveal phyla and ASVs that were disproportionally present in endospheres versus rhizospheres communities. In this model, plant compartment (i.e., endosphere vs. rhizosphere)—with samples from all sites—was treated as a fixed effect, and plant ID was included as a random effect. We visualised differential abundance of taxa using log‐fold changes, maintaining only statistically significant taxa at a 0.05 significance threshold.

We then conducted this analysis again, but for each individual sampling site separately, identifying taxa that are differentially abundant using the *ancombc* function across rhizospheres and endospheres within each of the eight sites separately. Following software instructions, this differential abundance testing was based on non‐rarefied data, and we used the false discovery rate for *p*‐value adjustment for multiple comparisons (Benjamini and Hochberg method) at both phylum and ASV levels of our data to identify differing taxa between endospheres and rhizospheres. We then compared sites to assess differences in rhizosphere‐to‐endosphere recruitment dynamics.

We then used a three‐step approach to explore the neutral and deterministic selection dynamics of the rhizosphere and endosphere differentially abundant ASVs. First, we isolated endosphere and rhizosphere ASVs from our dataset. Secondly, we created lists of ASVs that were disproportionately more abundant in the rhizosphere (=those with significant positive log‐fold change), those more abundant in the endosphere (=significant negative log‐fold change), and those that were not differentially abundant (non‐significant effect). Finally, we examined neutral assembly models based on these three lists of ASVs, using rarefied data for making model comparisons (as used in our diversity analyses), to separately assess selection in endosphere and rhizosphere communities. We used this approach to show how selection on ASVs changed from the rhizosphere to endosphere. Model fits were assessed using the coefficient of determination (*R* ^2^).

#### 2.5.4 Co‐Occurrence Network Analysis

We used co‐occurrence network analysis of bacterial ASVs to determine interactions between taxa and derive an indication of community structure within endospheres and rhizospheres. This analysis explores connections between different specific ASVs (i.e., nodes) via their correlative connections to one another (i.e., edges) by estimating significant positive or negative relationships between these taxa. We used SparCC to define absolute abundance associations between taxa at the ASV level, using the Spiec‐Easi R package (Friedman and Alm 2012; Kurtz et al. 2015).

For visualisations and computational processing of the network analyses, we only report ASVs with > 100 sequences. Randomly permuted (*n* = 1000) data were used to estimate the statistical significance of associations. Taxon associations were included using SparCC correlations at ≥ 0.65, with *p* < 0.05. We used the R package Matrix (Bates et al. 2023) to create a matrix from the given set of values and used igraph (Csardi et al. 2006) to visualise and evaluate the resulting networks. We identified ‘hub’ taxa as the top 30 bacterial ASVs with the highest number of positive or negative node edges (node degrees). These hub taxa, defined by the highest node degree, represent ASVs with the strongest and most numerous associations, indicating potential keystone roles in the community.

## 3 Results

### 3.1 Bacterial Diversity in Belowground Compartments

**FIGURE 2:**
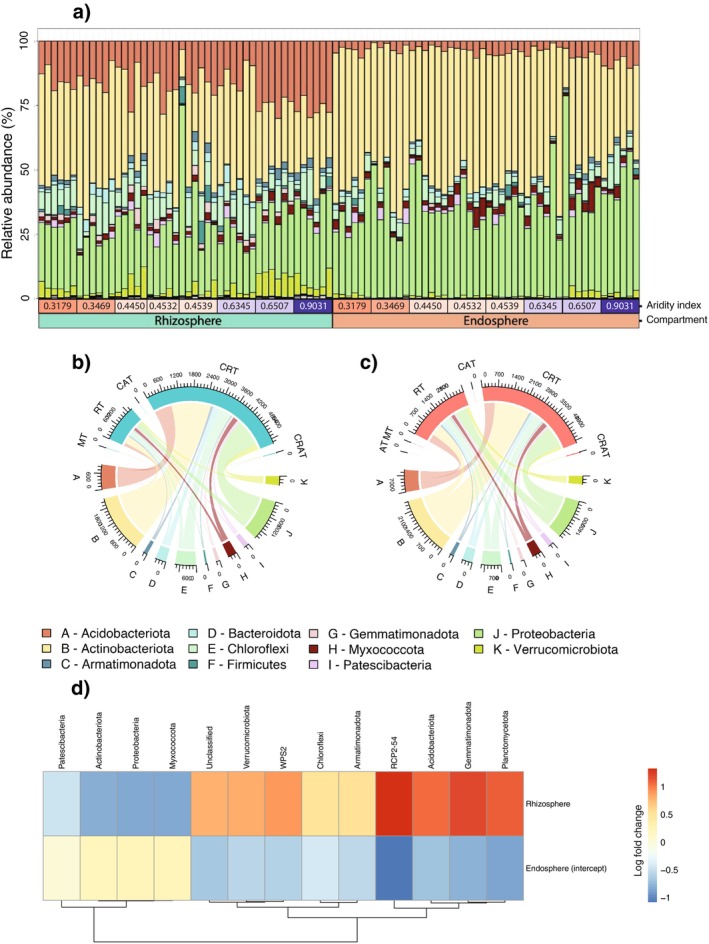
Bacterial ASV relative abundances visualised at the phylum level in endospheres and rhizospheres. (a) Stacked bars represent samples, grouped by aridity index of their sampling site. The bar colours represent the bacterial phylum. Chord diagrams for (b) rhizospheres and (c) endospheres, showing the relative proportion of each bacterial ASV within each phylum (groupings: A–K) found within bacterial abundance categories (AT—abundant taxa, MT—moderate taxa, RT—rare taxa, CAT—categorically abundant taxa, CRT—categorically rare taxa, and CRAT—categorically rare and abundant taxa). (d) Differential abundance analysis of major and minor bacterial phyla across the rhizospheres and endospheres.

Across all compartments (rhizospheres and endospheres), we observed 11 bacterial phyla that represented 99.5% of reads and had abundance estimates of > 2% (Figure 2a).

**FIGURE 3:**
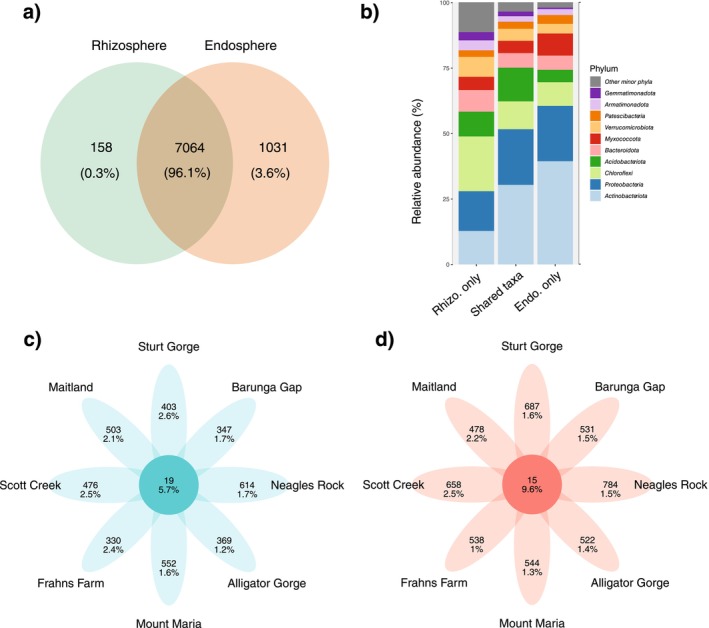
(a) Venn diagram of unique ASVs across *T. triandra* endospheres and rhizospheres showing the number of unique ASVs and the percentage of reads within each grouping, and (b) plot summarising relative abundance of phyla for the unique and shared ASVs in the endospheres and rhizospheres. (c) Partial Venn diagram showing unique *T. triandra* rhizosphere ASVs across each sampling site and shared across all sites, and the percentage of reads within each grouping; and (d) partial Venn diagram of unique ASVs across *T. triandra* endospheres in each site and shared across all sites, showing the number of unique ASVs and the percentage of reads within each grouping.

The alpha diversity of endospheres at the ASV level was 48.3% lower than rhizosphere diversity (effective number of ASVs was 153 in endospheres, versus 296 in rhizospheres) (LMM: *X* ^2^_(1)_ = 56.220, *p* < 0.001; Figure 1b), and there were many differences in alpha diversity across the sampling sites (LMM: *X* ^2^_(7)_ = 24.522, *p* < 0.001). Interestingly, there was no difference between the rhizospheres and endospheres using Faith's phylogenetic diversity (Figure S5: LMM: *X* ^2^_(1)_ = 0.7781, *p* < 0.378). Additionally, 96% of ASVs were shared by both compartments (Figure 3a), and 3.6% of taxa were unique to the endospheres (=1031 ASVs), whereas only 0.3% of taxa were unique to rhizospheres (=158 ASVs).

The composition of bacterial communities using Bray–Curtis dissimilarities for *T. triandra* was significantly different between endospheres and rhizospheres (Figure 1c; PERMANOVA: *F*_(1,76)_ = 10.888, *R* ^2^ = 0.078, *p* = 0.001, *n* = 48 samples per group). Furthermore, these communities were tightly clustered by sampling site (Figure S6; PERMANOVA: *R* ^2^ = 0.287, *F*_(7,76)_ = 5.741, *p* = 0.001, *n* = 6 per site). We also found that bacterial community composition changed with the aridity levels of our sampling sites across both the rhizospheres and endospheres (Figure S7; PERMANOVA: *R* ^2^ = 0.049, *F*_(1,90)_ = 4.626, *p* < 0.001).

We also found differences between rhizospheres and endosphere bacterial communities based on phylogenetically informed metrics: βMNTD (Figure S8a; PERMANOVA: *R* ^2^ = 0.078, *F*_(1,90)_ = 7.660, *p* < 0.001), and wunifrac (Figure S9a; PERMANOVA: *R* ^2^ = 0.034, *F*_(1,90)_ = 3.129, *p* < 0.001). Bacterial community composition also changed with differing site aridity using wunifrac distances (Figure S9b; PERMANOVA: *R* ^2^ = 0.065, *F*_(1,42)_ = 2.914, *p* < 0.001), and βMNTD (Figure S8b; PERMANOVA: *R* ^2^ = 0.040, *F*_(1,90)_ = 3.719, *p* < 0.001).

Endospheres were less varied than rhizospheres based on Bray–Curtis distances (Figure 2d; PemuTest: *F*_(1,90)_ = 36.24, *p* < 0.001), βMNTD (Figure S8c; PemuTest: *F*_(1,90)_ = 17.475, *p* < 0.001), and wunifrac distances (Figure S9c; PemuTest: *F*_(1,90)_ = 22.486, *p* < 0.001), suggesting reduced heterogeneity, and converging bacterial communities in *T. triandra* endospheres.

### 3.2 Taxonomic Rarity and Abundance

Bacterial ASVs were delineated into different rarity and abundance categories (see Section 2 for details; Table 2). Only one ASV was abundant in the endospheres across all samples (unidentified ASV belonging to the genus *Bradyrhizobium*, phylum: Proteobacteria), and no ASVs were abundant in the rhizosphere (> 1% abundance). As a proportion of the whole community, the greatest difference between endospheres and rhizospheres was in the CRT (< 1% in all sites, but < 0.01% in some), which comprised 62% of bacterial sequences in the endospheres (5070 ASVs) and 79% of sequences in the rhizospheres (5705 ASVs; Table 2). The RT (< 0.01% in all sites) also showed a large difference between compartments, comprising 37% of sequences in the endospheres (2970 ASVs) and 21% of sequences in the rhizospheres (1477 ASVs; Table 2).

**TABLE 2:** Bacterial ASVs allocated to six relative abundance categories.

Compartment	Category	Number of ASVs	Number of sequences
Rhizosphere~	Abundant taxa (AT)	0	0
	Moderate taxa (MT)	11 (0.15%)	7821 (1.55%)
	Rare taxa (RT)	1477 (20.45%)	5518 (1.09%)
	Conditionally abundant taxa (CAT)	4 (0.06%)	19,238 (3.81%)
	Conditionally rare taxa (CRT)	5705 (78.99%)	429,588 (84.97%)
	Conditionally rare and abundant taxa (CRAT)	25 (0.35%)	43,439 (8.59%)
Endosphere~	Abundant taxa (AT)	1 (0.01%)	13,094 (2.37%)
	Moderate taxa (MT)	6 (0.07%)	6755 (1.23%)
	Rare taxa (RT)	2970 (36.69%)	11,938 (2.16%)
	Conditionally abundant taxa (CAT)	4 (0.05%)	31,755 (5.76%)
	Conditionally rare taxa (CRT)	5070 (62.63%)	322,711 (58.51%)
	Conditionally rare and abundant taxa (CRAT)	44 (0.54%)	165,315 (29.97%)

Across the rhizospheres and endospheres, all ASVs from both communities had similar taxonomic compositions when considering their phyla across MT, CAT, and CRAT categories (Figure 2b,c). However, we did see a change in the relative number of bacterial sequences in the RT to CRT categories between rhizospheres and endospheres (Figure 2b,c).

### 3.3 Differential Abundance Among Endospheres and Rhizospheres

**FIGURE 4:**
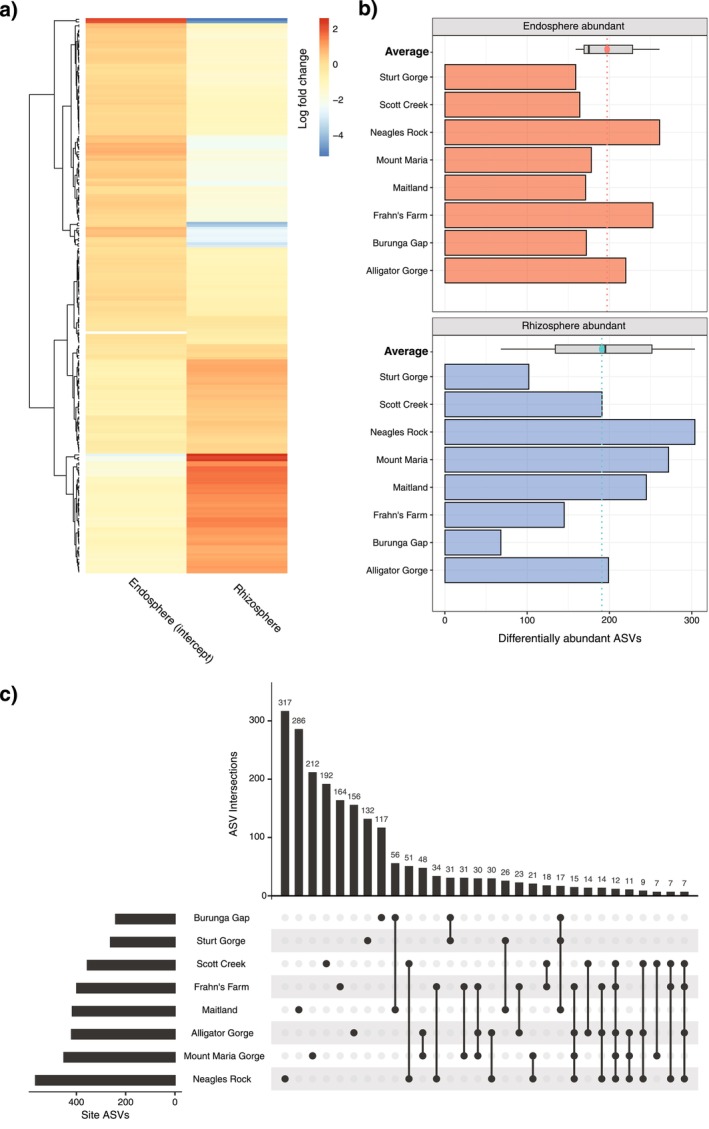
(a) Heatmap showing 218 differentially abundant ASVs across *T. triandra* rhizospheres and endospheres, with clustering of ASVs with high and low log‐fold changes represented by the dendrogram, and (b) the number of differentially abundant ASVs calculated within each sampling site. The negative grouping includes those ASVs favoured in endospheres (negative log‐fold change), whereas the positive grouping includes ASVs favoured in rhizospheres (positive log‐fold change). Sites are ordered from most to least arid (top to bottom, respectively). (c) Upset plot showing the number of shared and unique bacterial ASVs across each site. This plot shows the first 30 most populated ASV intersections between sites (see Figure S14 for all site intersections).

We found that 13 bacterial phyla were differentially abundant across the endospheres and rhizospheres using the ANCOM‐BC approach (analysis conducted at the phylum level: Figure S10a; Table S1). These were phyla with differences in abundance at the log‐fold‐change level across these two groups. For instance, phyla that were more abundant in the rhizosphere and reduced in the endosphere, and included bacteria attributed to Verrucomicrobiota, WSP2, Chloroflexi, Armatimonadota, RCP2‐54, Acidobacteriota, Gemmatimonadota, and Planctomycetota (Figure 2d). The endosphere abundant phyla included Patescibacteria, Actinobacteriota, Proteobacteria, and Myxococcota (Figure 2d). In a separate differential abundance analysis at the ASV level, we found that 218 ASVs were differentially abundant (Figure 4a; Table S2). Additional findings describing differentially abundant phyla at each site can be found in Figures S7, S11 and S12.

When we conducted this analysis at the ASV level for each site independently, we found 388 differentially abundant ASVs significantly different between endospheres and rhizospheres (Figure S13). We observed that 182 ASVs were more abundant in endospheres (negative log‐fold changes) and that 217 ASVs were more abundant in rhizospheres (positive log‐fold changes; Figure 4b). Interestingly, the differentially abundant ASVs between rhizosphere and endospheres at the site level were often unique to each site. Only one common ASV was differentially abundant between the rhizospheres and endospheres in every site, whereas a mean of 197 ASVs were uniquely differentially abundant across all sites (Figure 4c). The remaining ASVs were shared across two or more sites in various combinations, with diminishing counts as site‐site comparisons became more inclusive (Figures S13 and S14).

### 3.4 Selection of Microbiota Under Neutral Theory of Community Assembly

**FIGURE 5:**
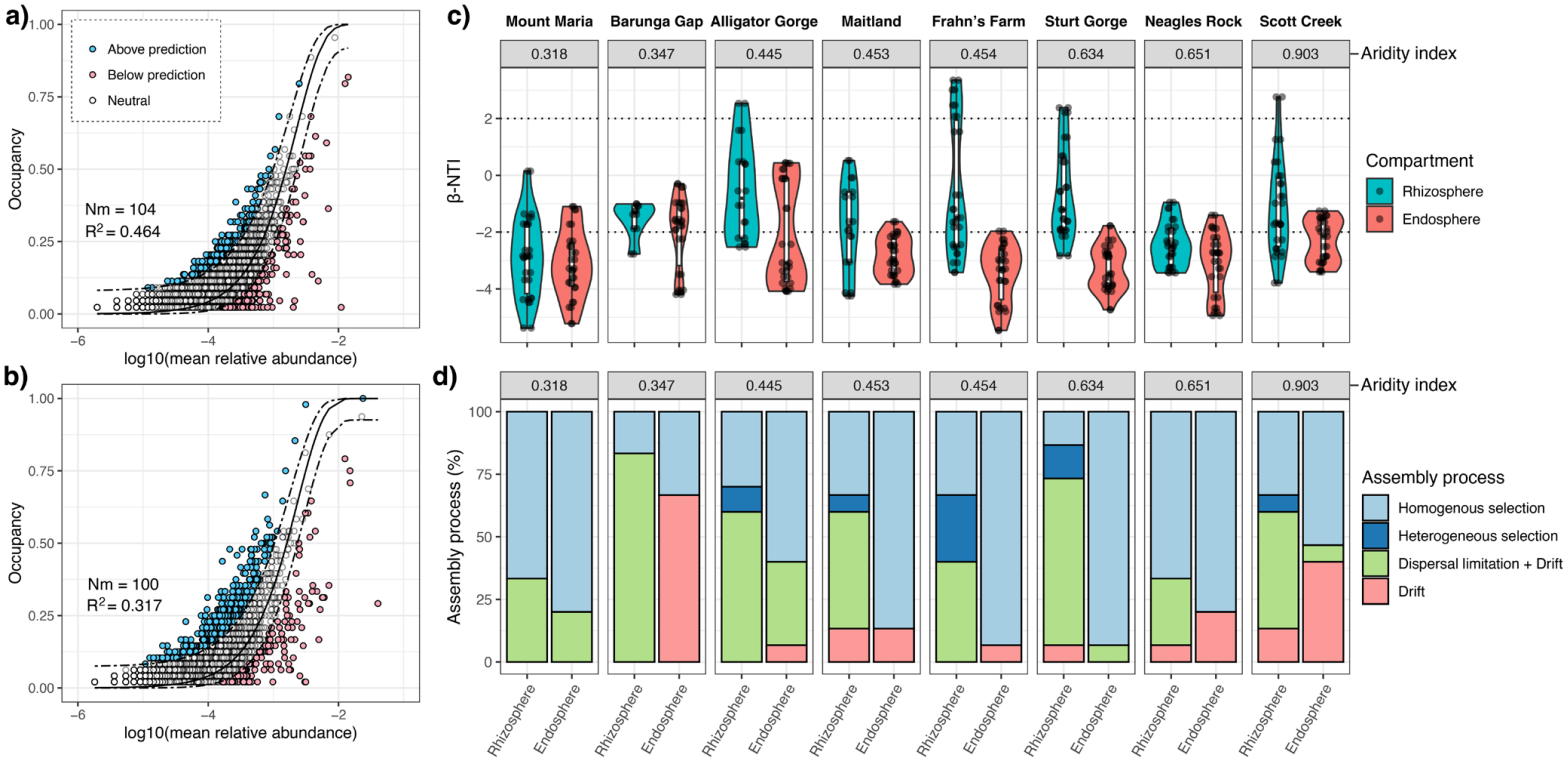
Abundance‐occupancy curves fitted with the Sloan neutral model in *T. triandra* (a) rhizospheres and (b) endospheres. Each point represents a bacterial ASV that occurs above (blue), below (pink), or within (white) neutral model predictions. ASVs that occur at greater occupancies than predicted by the neutral model (blue) are hypothesised to be positively selected by the environment, and those occurring with lower occupancies than predicted by the neutral model (pink) are hypothesised to be negatively selected by the environment. (c) βNTI values across the rhizospheres and endospheres of each sampling site in order of aridity index, and (d) stacked bar plot illustrating the relative contribution of ecological assembly processes across rhizospheres and endospheres in each sampling site in order of aridity index. Heterogeneous and homogeneous selection is attributed to βNTI values of > +2 or < −2, respectively. Communities without significant βNTI deviations (|βNTI | < 2) were investigated for homogenising dispersal or limiting dispersal with RCbray values of < −0.95 or > 0.95, respectively.

We fitted neutral assembly models to our rhizosphere and endosphere samples, including samples from all sites, to explore the neutral versus deterministic influences that shape their assembly. Endospheres fitted the neutral model to a lower degree than rhizospheres (Figure 5; endosphere, *R* ^2^ = 0.317, rhizosphere, *R* ^2^ = 0.464). This reveals a greater deterministic influence for shaping the selection of microbiota into the rhizosphere.

We then applied the neutral model to the different rarity and abundance categories of bacterial ASVs in rhizospheres and endospheres of all sites to explore differences in the assembly of the unique structural elements of the microbiota (i.e., rare and abundant taxa) (Figure S15). The abundance patterns of MT and CRT had a better fit to the neutral models in the rhizospheres (*R* ^2^ = 0.469, Figure S15a; *R* ^2^ = 0.49, Figure S15c, respectively) compared to the endospheres (*R* ^2^ = 0.12, Figure S15e; *R* ^2^ = 0.424, Figure S15g, respectively). The CRAT+CAT and RT neutral models had poorer fits in rhizospheres (albeit with RT producing a poor fitting model; *R* ^2^ = 0.295, Figure S15d; *R* ^2^ = −0.129, indicating failure to fit a model, Figure S15b, respectively), compared to the endospheres (*R* ^2^ = 0.175, Figure S15f; *R* ^2^ = 0.466, Figure S15h, respectively). Additional information on how the neutral models fit other subsets of our differential abundance analyses can be found in Figures S12 and S16.

In the rhizospheres and endospheres of each sampling site, we tested the influence of phylogenetic and bacterial community turnover to investigate the different deterministic and stochastic influences acting on these microbial communities. The endosphere microbiota were influenced by stronger selection pressures compared to the rhizospheres, with an average selection effect of 72.5% (±7.6% SE) for βNTI values > 2 or < −2, compared to 44.6% (±6.5% SE) for the rhizospheres (Figure 5c; Figure S17). Specifically, the endospheres were driven by homogeneous selection, which identified lower rates of phylogenetic turnover than expected under our null hypothesis (Figure 5d). Only rhizosphere communities were under any influence of heterogeneous selection (7.1% ± 3.4% SE), though they were mainly driven by homogeneous selection 37.5% (±7.1% SE). The dominance of stochastic processes also differed between rhizospheres and endospheres in the different sites. The effect of dispersal limitation plus drift (RCbray > 0.95), on average, was stronger in the rhizospheres 50.8% (±6.6% SE) than the endospheres 8.3% (±4.3% SE), but interestingly, the drift acting alone (RCbray ≥ −0.95, but ≤ 0.95) had, on average, a stronger influence on the endosphere communities 19.2% (±8.2% SE) than the rhizospheres 4.6% (±1.9% SE) (Figure 5d). We did not detect an influence of homogenising dispersal in the rhizospheres or endospheres (RCbray < −0.95).

### 3.5 Network Analysis and Hub Taxa

**FIGURE 6:**
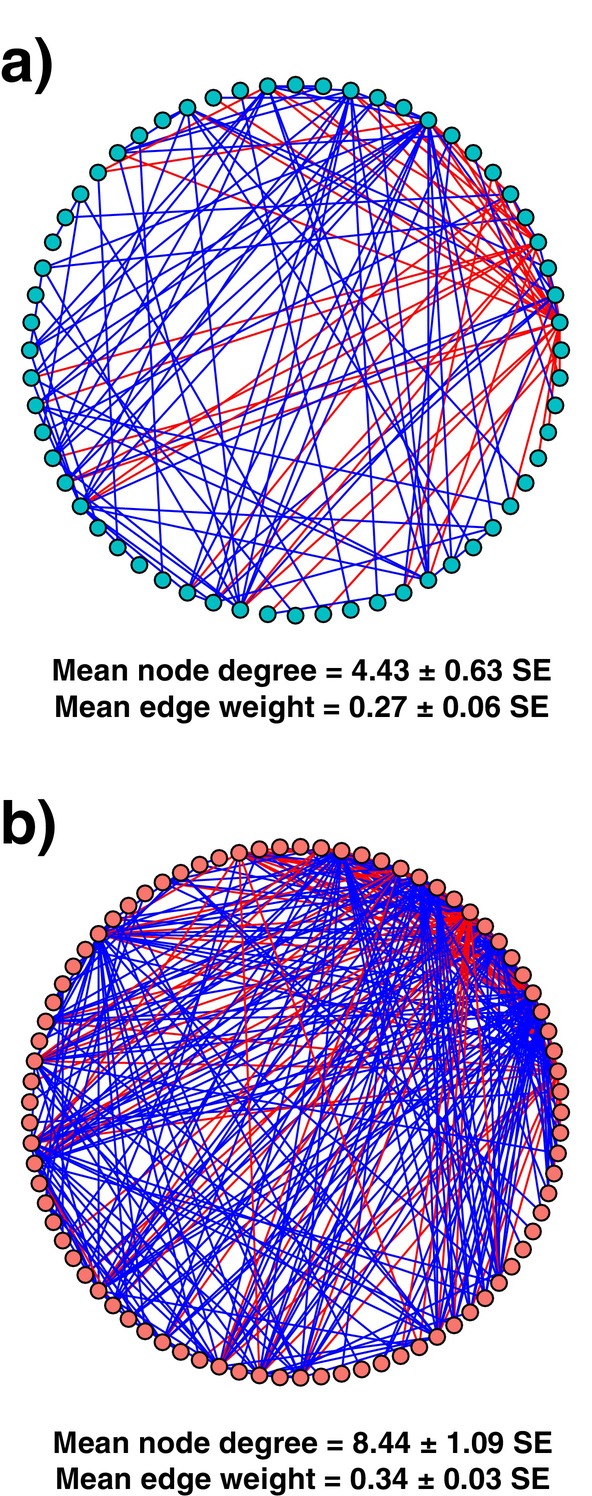
Network analysis of bacterial ASVs in (a) rhizospheres and (b) endospheres, sampled across *T. triandra* aridity gradient. Vertex colour indicates taxonomic groups at the ASV level. Positive associations are represented by blue edges, and negative associations are represented by red edges. The average degree and average edge weight are shown below for each network with their respective standard error.

Our network analysis included only associations between nodes (ASVs) with SparCC correlations ≥ 0.65 and *p*‐values < 0.05. We then removed any isolated nodes, resulting in endosphere networks with 81 nodes (ASVs) and rhizosphere networks with 60 nodes (Figure 6, Tables S3–S6). The rhizosphere networks had a lower average node degree (4.43 ± 0.63 SE vs. 8.44 ± 1.09 SE; Figure 6), indicating fewer significant associations between microbiota in rhizospheres compared to endospheres. Rhizospheres also showed lower average edge weight values (0.27 ± 0.06 SE vs. 0.34 ± 0.03 SE; Figure 6), suggesting more negative associations between taxa, while endospheres exhibited more positive associations among ASVs.

## 4 Discussion

We investigated the patterns of microbial assembly in rhizospheres and root endospheres in natural populations of the non‐model pan‐palaeotropical C4 grass species, *Themeda triandra* . We found that rhizosphere and endosphere diversity patterns were consistent with the second step of the two‐step selection process (Bulgarelli et al. 2013)—endospheres were less diverse than rhizospheres. We also observed convergence in endospheres across populations, where these bacterial communities were more homogeneous than rhizospheres. Despite this convergence, endosphere recruitment was also influenced by site‐specific factors, including aridity (with differences among bacterial community compositions across the gradient). We found more unique bacterial ASVs in endospheres than rhizospheres, which suggests a potential role of vertical transmission (i.e., parent to offspring transfer) and/or life stage dependency on endosphere colonisation. Finally, we found that assembly processes in endospheres had a stronger deterministic influence than in rhizospheres, and that there was a core microbiome (taxa that persistently occur within a given environment) in these endospheres that likely supports the functioning of *T. triandra* . A deeper understanding of these microbial interactions would help inform plant and soil resource management during conservation and restoration efforts (e.g., propagation, translocation, revegetation).

### 4.1 Two‐Step Selection Process

We observed that overall bacterial diversity in *T. triandra* endospheres was approximately 48% lower than in rhizospheres. Both endospheres and rhizospheres had distinct community compositions from each other, which is consistent with expectations under the two‐step selection process. Our diversity and composition findings are in line with previous work on *Arabidopsis thaliana* , where several studies have demonstrated the selection of microbiota across soil and rhizosphere environments into root endospheres (Bulgarelli et al. 2013; Urbina et al. 2018; Barajas et al. 2020). These previous studies suggest that the controlled release of exudates by the plant attracts and supports the recruited microbiota (Bai et al. 2022). Though we did not directly measure root exudates, we do find compelling results from a bacterial community perspective, which is supported by our previous *T. triandra* soil–rhizosphere study (Hodgson et al. 2024). Here, we present strong evidence that the two‐step selection process outlined by Bulgarelli et al. (2013) is active in the rhizospheres and endospheres of this non‐model, keystone grass species. Importantly, we report these results from naturally occurring populations of this grass species, which is a noteworthy difference from previous studies, which generally focused on plants growing *ex situ* and under controlled lab or greenhouse conditions.

Our detailed investigation into assembly patterns revealed stronger deterministic processes in the endospheres compared to the rhizospheres, which is consistent with the general assumptions of the two‐step selection process—the host plant is expected to exert greater regulatory and selective control over microbiota entering the roots than over those in the rhizosphere (Bulgarelli et al. 2013). Additionally, the endospheres also contained more ASVs that deviated from the neutral theory model than the rhizospheres, and homogeneous selection processes were dominant for explaining the assembly of endosphere communities. As such, endospheres recruit phylogenetically similar bacterial communities, likely for distinct roles that target similar bacterial traits according to the eco‐physiological needs of the host plant or required to pass host immune system filtering (Stegen et al. 2012; Zhang et al. 2021; Wang et al. 2023).

Alongside the homogeneous selection acting on the endosphere communities, we found a stronger than expected influence of ecological drift (random population changes) on the endosphere microbial communities. Microbe‐microbe interactions, specifically priority effects, may explain this as early colonisers likely create conditions in the endospheres that facilitate the establishment of other species (Rillig et al. 2015). These interactions could alter the endosphere environment through resource competition and/or metabolic processes that limit the strength of host‐imposed selection processes. As such, stochastic events—such as random fluctuations in microbial populations—can play a larger role in community assembly than expected in such a regulated environment (Rillig et al. 2015; Debray et al. 2022). In contrast to the endospheres, the rhizosphere microbiota were more strongly influenced by dispersal limitation (low dispersal rates) compared to ecological drift alone (Stegen et al. 2013). The low community turnover in the rhizospheres suggests that these bacterial communities were strongly shaped by the diversity and dispersal potential of local soil microbiota (Zhang et al. 2021). This highlights the constraining influence of local conditions on the two‐step selection process. Future work should focus on identifying the root exudates involved and assessing the fitness consequences of these assembly processes by characterising the functional processes of microbiota involved.

### 4.2 Endosphere Convergence

We report that local site conditions influenced endosphere recruitment dynamics, which resulted in a unique assortment of differentially abundant bacterial ASVs in the endospheres across sites—an effect also observed in previous studies on the trees *Populus deltoides* and *Taxodium distichum* (Gottel et al. 2011; Lumibao et al. 2020). Observing different endospheres across sites suggests that either stochastic effects, local conditions and/or resource availability affected how *T. triandra* regulates inbound microbiota (Vandenkoornhuyse et al. 2015). These influences were consistent with our earlier work which showed that *T. triandra* bulk soil microbial communities and rhizospheres were strongly shaped by soil nutrient levels, aridity and moisture availability (Hodgson et al. 2024). Local conditions are well known to shape bulk soil, rhizospheres and endospheres, however, in our study, these site‐specific effects did not appear to impede the development of a convergent root endosphere across populations. Factors that shape internal microbial profiles could also shape preferential niches created by the host plant or some combination of other influences, such as microbe‐mediated priority effects (Rillig et al. 2015). This raises intriguing questions about the functional potential of the ‘core’ microbial endosphere, and follow‐up studies should investigate this further.

We reported higher overall complexity (based on node degree in our network analysis) and positive associations of ASVs in endospheres compared to rhizospheres, indicating remarkable symbiosis inherent in the convergence of bacterial communities in these root compartments. The top connected ASVs (=hub taxa) are often hypothesised to be keystone species that support or facilitate the recruitment of other microbiota (Rillig et al. 2015; Trivedi et al. 2020; Debray et al. 2022). Additionally, a decrease in the ratio of CRT to RT within rhizospheres compared with endospheres (1.71 vs. 3.84, respectively) shows that rhizospheres often support highly varied microbial community structures that are also more diverse (i.e., greater alpha diversity). As expected, we report new evidence of bacterial symbioses in *T. triandra* endospheres (i.e., less influenced by local soil and/or climatic conditions) relative to rhizospheres, which supported ASVs with fewer key microbe‐microbe associations (Trivedi et al. 2020).

### 4.3 Vertical Transmission of Microbiota

The high count of bacterial ASVs that were unique to root endospheres were likely populated via vertical transmission (i.e., from parent plant flowers to their offspring during seed development) (Bulgarelli et al. 2013; Escobar Rodríguez et al. 2018; Abdelfattah et al. 2023); or transferred across host compartments (e.g., leaves or stems into roots; Chi et al. 2005). There is strong evidence of vertically transmitted bacterial endophytes being involved in mobilising plant nutrients and affecting phytohormone signalling inside roots (Bulgarelli et al. 2013; Santoyo 2022). Future research should explore whether unique ASVs within each site are inherited through vertical transmission due to local adaptation of *T. triandra* populations (Thiergart et al. 2020; Durán et al. 2022). As such, this form of parent to offspring transfer could be important to *T. triandra* fitness, where microbiota cannot survive independently in soil environments and host plants may have evolved traits that facilitate the persistence of a portion of the microbial community (Johnston‐Monje and Raizada 2011; Lumibao et al. 2020; Lyu et al. 2021).

It is worth considering that the ASVs suspected of vertical transmission in this study could still be a product of the two‐step selection process, especially if we simply did not observe them in the rhizosphere during sequencing due to insufficient sequence depth or the changing nature of rhizospheres across plant developmental stages. Further research could investigate how horizontally transferred bacterial taxa (i.e., soil to root endosphere colonisation) are supported in soil environments and whether they require their plant hosts for completion of their life cycles (i.e., are they obligate symbionts?) (Vandenkoornhuyse et al. 2015). These ASVs may have a dormant, protected life stage (e.g., spore‐forming) (van Vliet 2015), or could perhaps be microbiota that are influenced by host plant demographics and local adaptation (Ledeganck et al. 2003; Hannula et al. 2021). Further investigations should consider the vertical transmission of root endospheres.

## 5 Conclusions

We show that the microbiomes of natural populations of *T. triandra* growing across diverse environments retain assembly processes consistent with root endosphere colonisation from rhizospheres. We show that deterministic assembly processes acted strongly on these endospheres, as they were strongly affected by both environmental factors (e.g., aridity) plus host selection for similar microbial communities and traits within sampling sites (homogeneous selection). Additionally, while numerous endosphere taxa were likely from the plant rhizospheres, we present evidence for probable vertical transmission of microbiota from parent to offspring. Our limited understanding of the complex roles of plant‐associated microbiota hinders our ability to harness the ecology of these important relationships in an applied ecology context (e.g., propagation, translocation, revegetation). Future investigations should consider the functional roles and inheritance patterns of root endosphere microbiota in non‐model plant species, and assess how these plant‐microbe interactions affect host fitness.

## Author Contributions

**Riley J. Hodgson:** conceptualization (lead), formal analysis (lead), methodology (lead), visualization (lead), writing – original draft (lead), writing – review and editing (lead). **Christian Cando‐Dumancela:** data curation (equal), investigation (equal), writing – review and editing (equal). **Craig Liddicoat:** formal analysis (equal), methodology (equal), writing – review and editing (equal). **Sunita A. Ramesh:** conceptualization (equal), formal analysis (equal), investigation (equal), methodology (equal), writing – review and editing (equal). **Robert A. Edwards:** writing – review and editing (equal). **Martin F. Breed:** conceptualization (equal), funding acquisition (equal), methodology (equal), resources (equal), supervision (equal), writing – original draft (equal), writing – review and editing (equal).

## Conflicts of Interest

The authors declare no conflicts of interest.

## Supporting information


Data S1.

## Data Availability

All data supporting the findings of this study have been made publicly available on Figshare (DOI: 10.25451/flinders.27239697). Raw sequence data have been deposited in the NCBI Sequence Read Archive under accession numbers PRJNA1029310 and PRJNA1138818.
