# Supplementary material for: Strong Host Modulation of Rhizosphere‐to‐Endosphere Microbial Colonisation in Natural Populations of the Pan‐Palaeotropical Keystone Grass Species, Themeda triandra
Source: Ecol Evol. 2025 Jun 18;15(6):e71595. doi: 10.1002/ece3.71595 (PMC12174961; doi:10.1002/ece3.71595)
Supplement: Supplementary file 1 — Data S1. [file ECE3-15-e71595-s001.docx]

# **Supplementary information**

# **Strong host and environment modulation of rhizosphere-to-endosphere microbial colonisation in the pan-palaeotropical keystone grass species, *Themeda triandra***

Riley J. Hodgson^1^, Christian Cando-Dumancela^1^, Craig Liddicoat^1^, Sunita Ramesh^1^, Robert Edwards^1^ & Martin F. Breed^1^

^1^College of Science and Engineering, Flinders University, Bedford Park, SA 5042, Australia.

**Corresponding author**: Riley J. Hodgson, College of Science and Engineering, Flinders University, Bedford Park, SA 5042, Australia, +61 (0)8 8201 2113, [riley.hodgson@flinders.edu.au](mailto:riley.hodgson@flinders.edu.au)

**ORCIDs**: RJH 0000-0002-8043-2473, CCD 0000-0003-0186-0056, CL 0000-0002-4812-7524, SR 0000-0003-2230-4737, RE 0000-0001-8383-8949 , MFB 0000-0001-7810-9696

**SUPPLEMENTARY METHODS**

*Pilot experiment*

In March 2022, endospheres from *T. triandra* plants were obtained from 15 individuals at Flinders University (35° 1' 42.95", 138° 34' 37.38") to test the efficacy of different root cleaning methods to isolate endosphere microbiota (n=3). Endosphere microbe isolation is a common procedure undertaken across a variety of different plants, often studies employ sonication of the plant roots to remove surface microbiota, or chemical sterilisation with solutions such as NaOCl, for instance (Barra et al. 2016, Richter-Heitmann et al. 2016). These methods have been published for a host of model species (Bulgarelli et al. 2013, Urbina et al. 2018), outcomes and effectiveness, however, could differ depending on the species of plant and root types. For *T. triandra* plants obtained under field conditions, we tested four root washing treatments: sterilisation of root surfaces by submerging in either 2% or 4% NaOCl for a period of 3 minutes; sonication by probe in 0.02% Silwet L-77 amended PBS buffer for either 3 minutes or 5 minutes, each in 30 second burst and rest periods; and a wash only control treatment in the amended PBS buffer. Following these cleaning processes, plant roots were subsequently washed three times in 0.02% Silwet L-77 amended PBS buffer solution. During the final wash step a 100 uL samples was taken for each sample and plated on LB (Luria-Bertani Agar), and placed in an incubator at 28°C. Root samples were subsequently prepared for DNA extraction, in anticipation for amplicon sequencing of the 16S rRNA gene to identify bacteria present.

**SUPPLEMENTARY RESULTS**

*Pilot study*

Following bioinformatic processing, we show that diversity was reduced in the bleached root samples, significantly below that of sonication and the control group (Figure S1a). Interestingly, the community composition did not appear to change much with sonication and control group, though there was a significant shift with the bleached treatments at 2% and 4% (Figure S1b-c).

Microbial colonies present on Luria Bertani agar showed different results following each the root cleaning processes. Results found that 2-4% bleach was the most effective process for removing contaminant taxa from root surfaces, followed 5 min sonication treatment compared to the control groups (Figure S2a). Log transformed concentrations of DNA extracted from root tissue across different cleaning methods (Figure S2b). Both bleach treatments appeared to have too great an effect on removing DNA from root samples, additionally removing DNA from the internal root structures, whereas in sonication treatments concentrations remained high, in similar quantities to the control group. Sonication of the roots for 5 minutes (30 second burst and rest periods) was identified as the most appropriate methods for use on *T*. *triandra* sampled roots based on our sampling procedure. To fine-tune sonication methods before applying this approach to samples from the field experiment, we explored the effect of increasing the number of different 0.02% Silwet L-77 amended PBS buffer wash steps before extraction. Additional wash steps improved the cleaning of the roots, as shown by the number of colonies identified on LB agar plates with the fewest colonies shown after 5 washes (Figure S2b).

*Differential abundance analysis of ASVs and Phyla with neutral model fits*

When we performed the differential abundance analyses separately for at each site, and compared site outcomes together, we found an average of 8.5 (±0.98 SE) differentially abundant phyla were present across rhizospheres and endospheres (Figure S7b). Specifically looking at directional trends, we found that 4.5 (±0.5 SE) phyla were more abundant in the endospheres (negative log fold changes) and 4 (±0.8 SE) phyla were more abundant in rhizospheres (positive log fold changes; Figure S11; Figure S12).

The ASVs that were differentially abundant between the *T. triandra* rhizospheres and endospheres were also tested with neutral assembly models, and revealed to be differently impacted by microbial community assembly selection dynamics (Figure S16). Differentially abundant rhizosphere-favoured ASVs (those with a significant positive log fold change, see Main document, Figure 4a) displayed a better fit to the neutral models in rhizospheres (R^2^ = 0.286; Figure S16a), compared to endosphere abundant taxa (negative log fold change, see Main document, Figure 4a) (R^2^ = 0.014; Figure S16e). ASVs that were not differentially abundant between the rhizospheres and endospheres still collectively deviated from the neutral models, suggesting that deterministic processes were influencing many taxa in these compartments. This pattern was consistent across compartments, with a similar deviation from neutral models measure in the non-differentially abundant ASVs in rhizospheres (R^2^ =0.039, Figures S16c) as those in the endospheres (R^2^ =0.028, Figure S16f).

When looking at how the neutral models fitted the endosphere-favoured ASVs (negative log fold change) within the rhizosphere samples (i.e., low abundance rhizosphere taxa), we observed strong neutral influences (R^2^ = 0.695, Figure S16b); and similarly, the rhizosphere-favoured ASVs (positive log fold change) when found in endosphere samples (i.e., low abundance endosphere taxa) saw comparatively strong neutral influences (R^2^ = 0.454, Figure S16d).

**FIGURES**

**
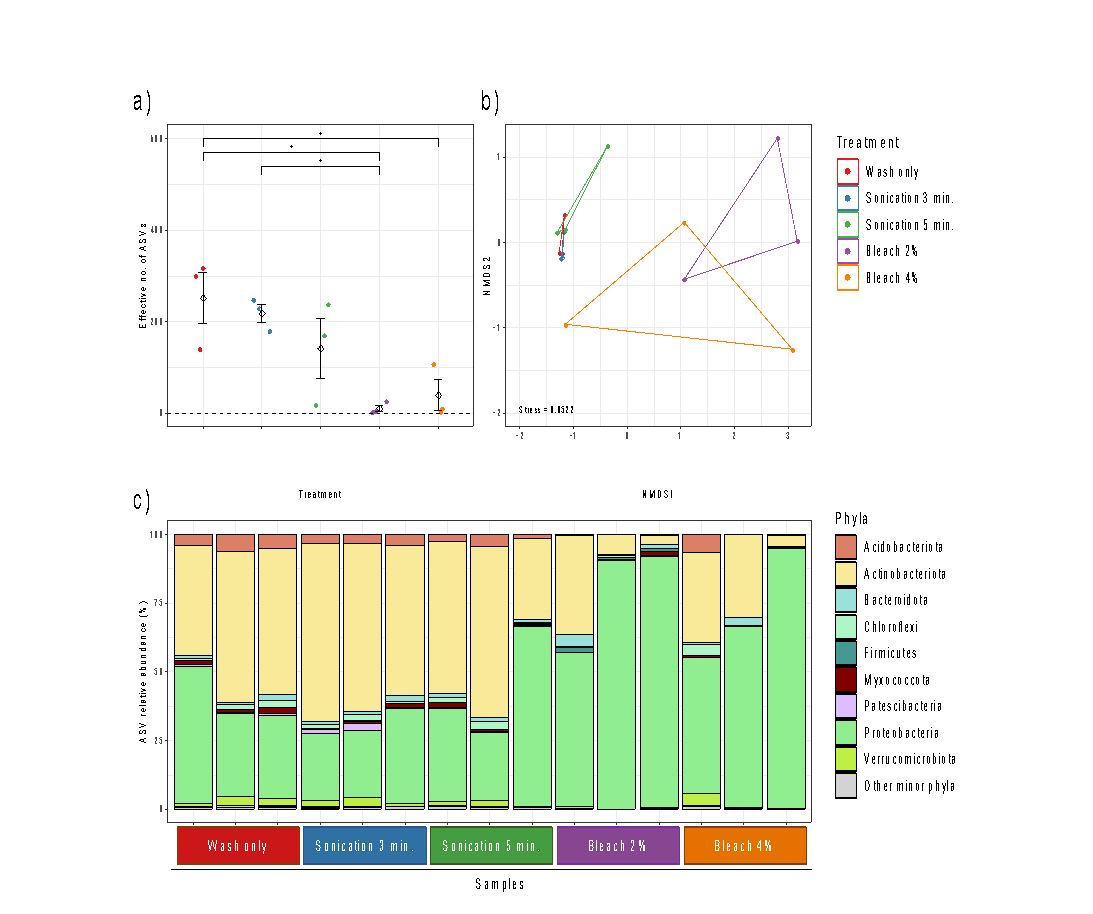
**

**Figure S1.** Root cleaning methods to isolate endosphere microbiota in *T. triandra* individuals. (a) bacterial diversity as effective number of ASVs, against chemical and mechanical cleaning methods. (b) NMDS ordination showing the effect of diferent cleaning methods on bacterial community composition. Polygons are coloured by treatment group. (c) Relative abundance of major bacterial phyla across samples and cleaning treatments.

**Figure S2.** (a) Microbial colonies present on Luria Bertani agar following different root cleaning processes. Results found that 2-4% bleach was the most effective process for sterilising root surfaces, followed 5 min sonication, compared to the control groups. (b) Log transformed concentrations of DNA extracted from root tissue across different cleaning methods. Bleach appeared to have too great an effect on removing DNA from root samples, whereas sonication concentrations remained high, in similar quantities to the control group.

**Figure S3.** After establishing sonication as the most effective root cleaning method to isolate endosphere microbiota, with most sterilised wash mediums and best DNA yields. We compared the number of wash steps in 0.02% Silwet L-77 amended PBS, following primary cleaning of the roots to limit the formation of bacterial colonies.

**Figure S4.** Rarefaction plot showing number of ASVs by number of reads per sample. Samples were rarified to 11,491 reads (red dashed line). Samples that did not meet the minimum threshold of reads were removed from analysis.

**Figure S5.** Faith’s phylogenetic diversity for rhizospheres and endospheres communities.

**Figure S6.** Non-metric multi-dimensional scaling **(**NMDS) ordinations with Bray-Curtis distances showing the difference between sampling sites across (a) endosphere samples (stress = 0.0922) and (b) rhizosphere samples (stress = 0.1200). Site are represented by colour.

**Figure S7.** Non-metric dimensional scaling plot with Bray-Curtis distances showing the difference between plant compartments. Endosphere samples represented by circles, and rhizospheres by triangles. Mean annual aridity index of sampling sites is indicated by colour gradient with a lower aridity index values corresponding to higher site aridity estimates.

**Figure S8.** (a) Non-metric dimensional scaling plot with beta mean nearest taxon distances (bMNTD) showing the differences between bacterial community composition across plant rhizospheres (blue) and endospheres (red). (b) NMDS plot with beta mean nearest taxon distances (bMNTD) showing the differences between bacterial community composition. Endosphere samples represented by triangles, and rhizospheres by circles. Mean annual aridity index of sampling sites is indicated by colour gradient with a lower aridity index values corresponding to higher site aridity estimates. (c) Distance to centroid of samples comparing rhizosphere (blue) and endosphere (red) samples, calculated from bMNTD.

**Figure S9.** (a) Non-metric dimensional scaling plot with weighted unifrac distances showing the differences between bacterial community composition across plant rhizospheres (blue) and endospheres (red). (b) NMDS plot with weighted unifrac distances showing the differences between bacterial community composition. Endosphere samples represented by triangles, and rhizospheres by circles. Mean annual aridity index of sampling sites is indicated by colour gradient with a lower aridity index values corresponding to higher site aridity estimates. (c) Distance to centroid of samples comparing rhizosphere (blue) and endosphere (red) samples, calculated from weighted unifrac distances.


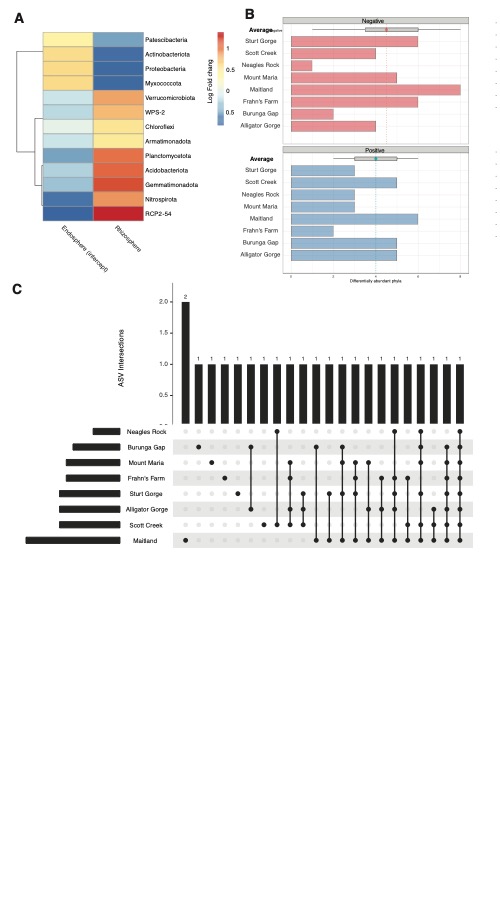


**Figure S10.** (a) Heatmap showing 13 differentially abundant bacterial phyla across *T. triandra* rhizospheres and endosphere samples and (b) the number of differentially abundant phyla calculated within each sampling site. The negative grouping includes those phyla favoured in the endosphere (negative log fold change), whereas the positive grouping includes phyla favoured in the rhizosphere (positive log fold change). (c) Upset plot showing the number of shared and unique bacterial phyla across each site that are differentially abundant. This plot shows only the first 22 most populated ASV intersections between sites (see Figure 10 for full figure).

**Figure S11.** Upset plot showing the number of shared and unique bacterial phyla across each sampling site that are differentially abundant.


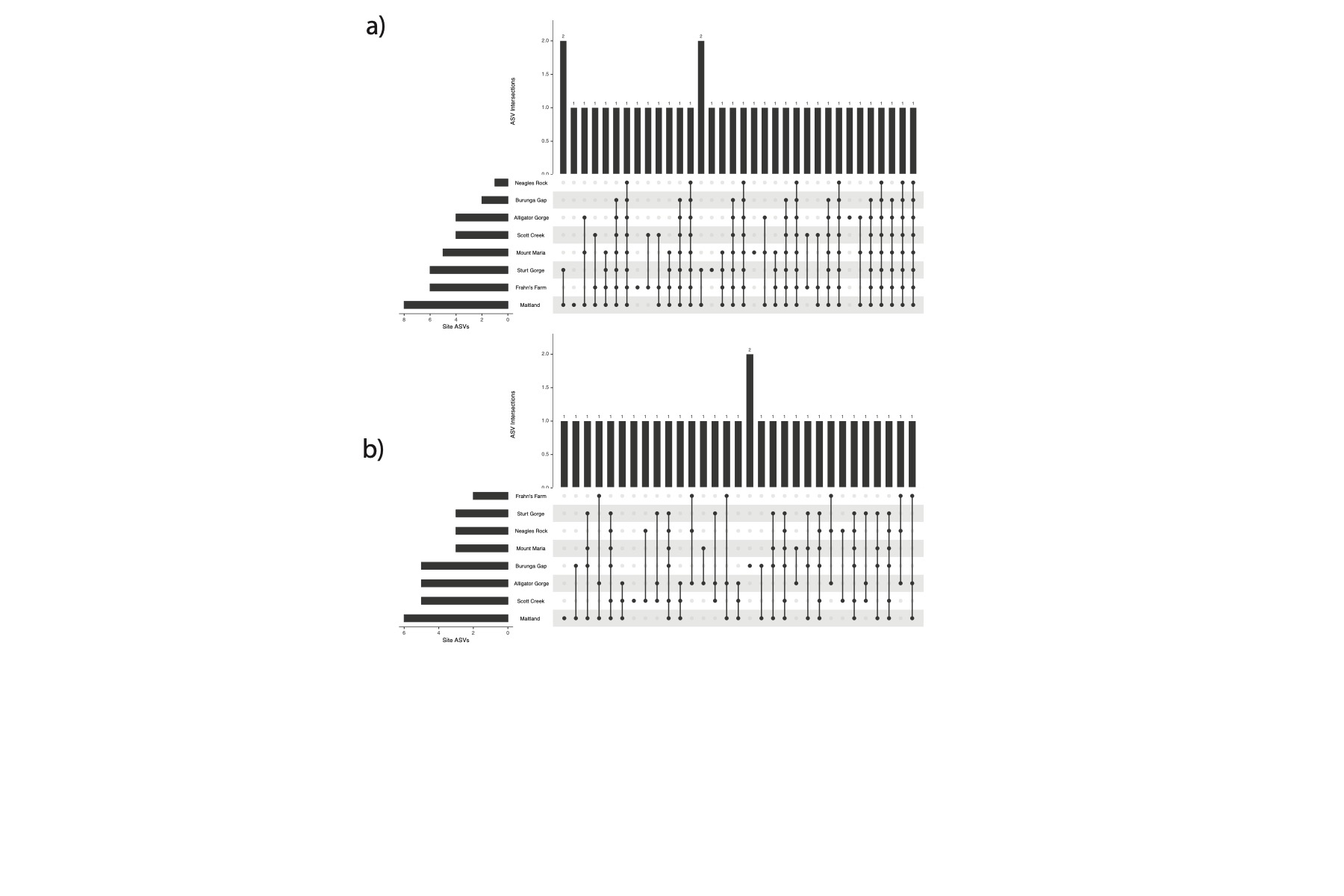


**Figure S12.** Upset plot showing the number of shared and unique bacterial phyla across each sampling site that are differentially abundant with either a (a) negative and (b) positive log fold change in the rhizosphere relative to the endosphere

**Figure S13:** Upset plot showing the number of shared and unique bacterial ASVs across each sampling site that are differentially abundant. The order of the bars representing site intersections (overlapping ASVs between sites) are ordered first by the sites with the highest to lowest total number of ASVs (left panel bar), and then within sites by the groupings from highest to lowest intersection counts with other sites.


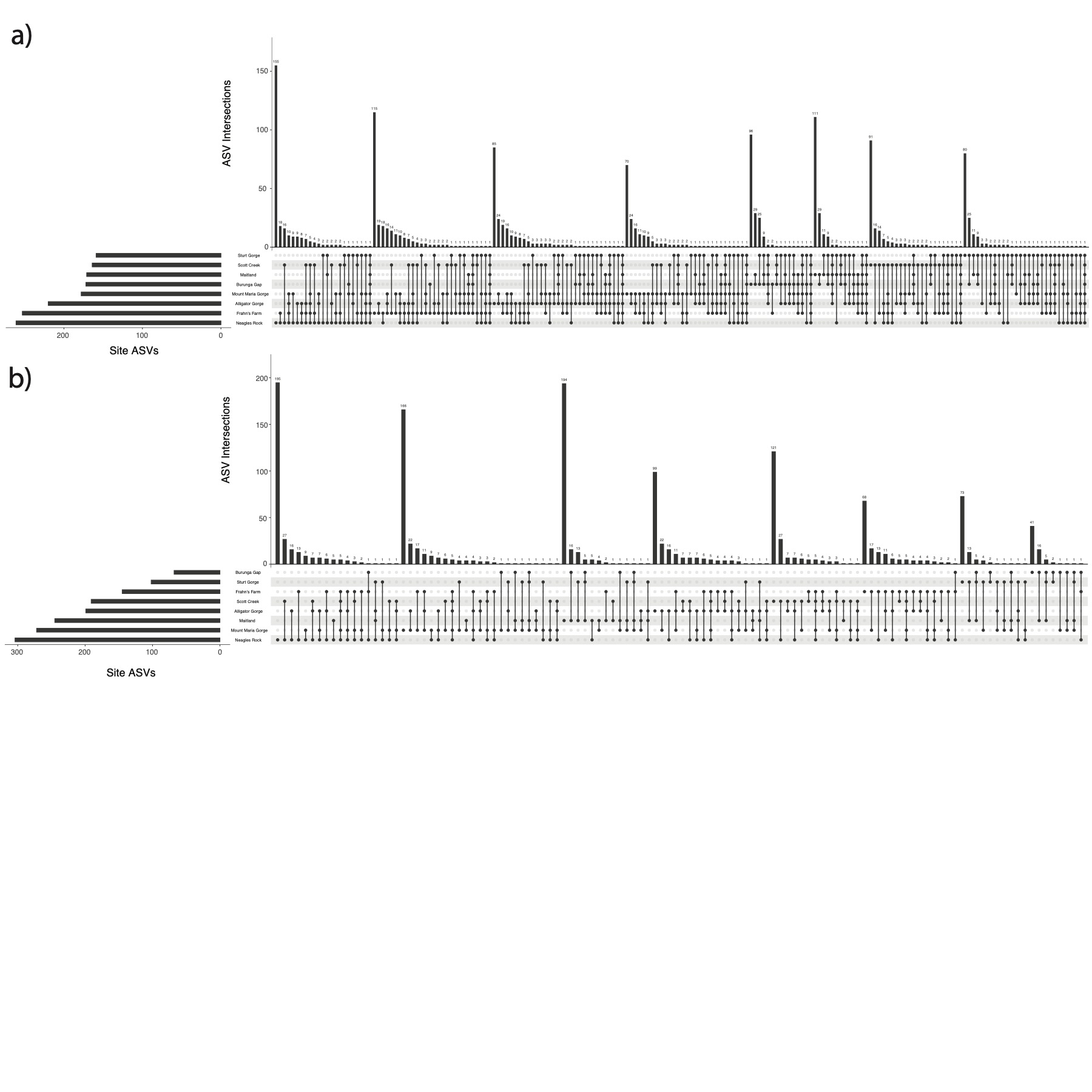


**Figure S14:** Upset plot showing the number of shared and unique bacterial ASVs across each sampling site that are differentially abundant with either a (a) negative and (b) positive log fold change in the rhizosphere relative to the endosphere. The order of the bars representing site intersections (overlapping ASVs between sites) are ordered first by the sites with the highest to lowest total number of ASVs (left panel bar), and then within sites by the groupings from highest to lowest intersection counts with other sites.

**Figure S15.** Abundance-occupancy curves fitted with Sloan neutral model in *T. triandra* (a-d) rhizospheres and (e-h) endospheres. Each point represents a bacterial ASV that was categorised as moderate taxa (MT, orange; panels a and e), rare taxa (RT, blue; panels b and f), conditionally rare taxa (CRT, pink; panels c and f), and the conditionally rare and abundant taxa plus the conditionally abundant taxa (CRAT+CAT, yellow and green, respectively; panels d and h), against a neutral model (black line) with 95% confidence intervals (dashed lines). The negative coefficient of determination for rare taxa (panel b) indicates failure to fit a model.

**
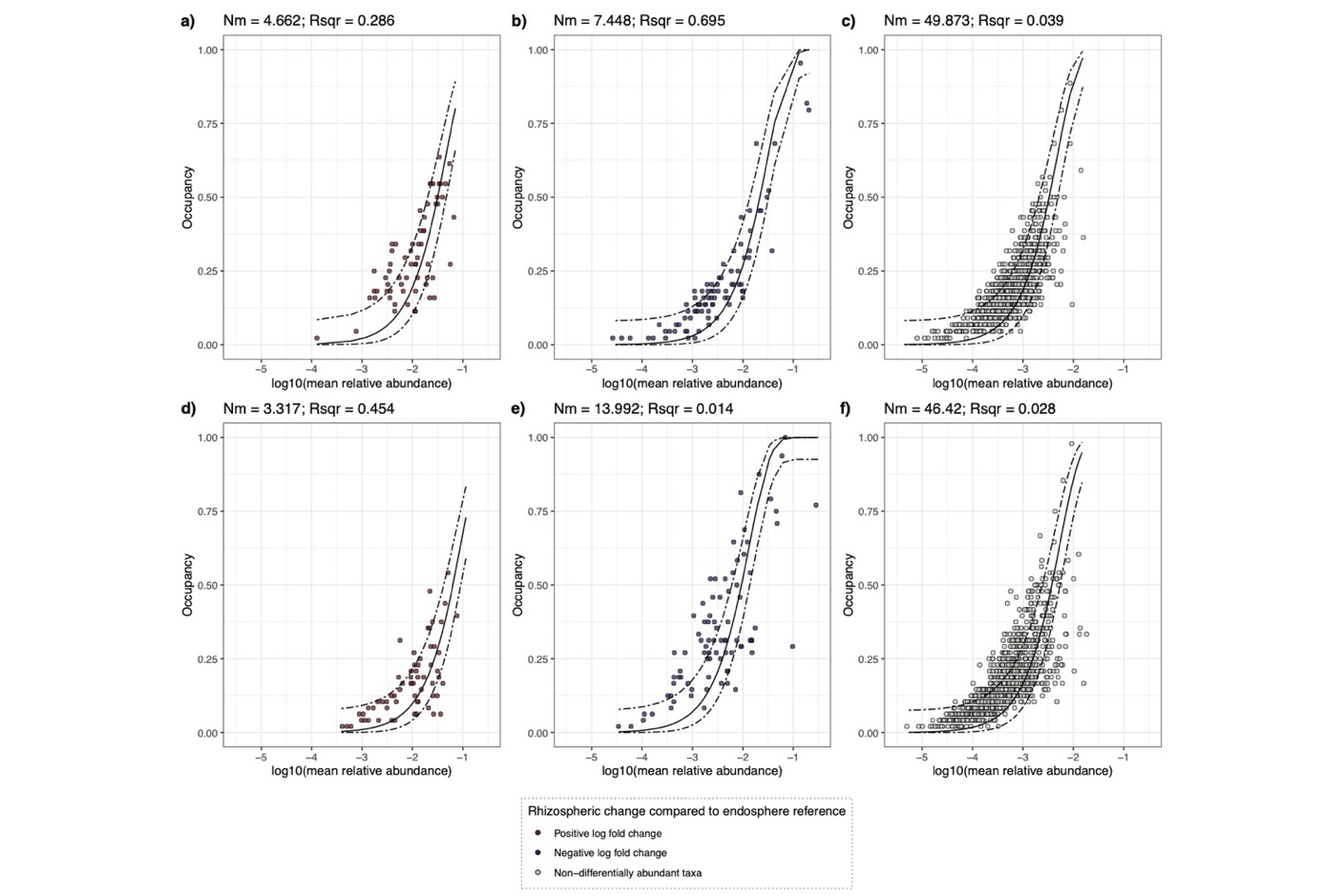
**

**Figure S16.** Abundance-occupancy curves fitted with Sloan neutral model in *T. triandra* (a-c) rhizosphere only samples and (d-f) endosphere only samples. Each point represents a bacterial ASV that was either differentially abundant with positive log fold change in the rhizosphere (rhizosphere-favoured, red; a and d), those with a negative log fold change compared to the rhizosphere (endosphere-favoured, blue; b and e), or those that were not differentially abundant (white; c and f). Each was plotted against a neutral model (black line) with 95% confidence intervals (dashed lines).

**Figure S17.**  βNTI values for rhizospheres and endosphere within each site (See main document Figure 5c), but pooled for simpler interpretation. Aridity index values indicate the aridity level for each of the sampling sites (0.318-0.907), where low aridity index indicates drier conditions, and high aridity index indicates wetter conditions. Heterogeneous and homogeneous selection is attributed βNTI values of > +2 or < -2, respectively. Communities without significant βNTI values (|βNTI | <2) indicate the influences of stochastic processes on microbial community assembly.

**TABLES**

**Table S1:** Differentially abundant bacterial phyla with positive or negative magnitude of change across in the rhizospheres relative to root endosphere taxa. Data includes all differentially abundant phyla with a positive or negative direction of change, and log fold change.

| Phylum | Direction | Log fold change |
| --- | --- | --- |
| RCP2-54 | Increasing | 1.367 |
| Gemmatimonadota | Increasing | 1.172 |
| Acidobacteriota | Increasing | 1.1 |
| Planctomycetota | Increasing | 1.056 |
| Nitrospirota | Increasing | 0.911 |
| Verrucomicrobiota | Increasing | 0.853 |
| WPS-2 | Increasing | 0.762 |
| Chloroflexi | Increasing | 0.545 |
| Armatimonadota | Increasing | 0.509 |
| Patescibacteria | Decreasing | -0.531 |
| Actinobacteriota | Decreasing | -0.75 |
| Proteobacteria | Decreasing | -0.772 |
| Myxococcota | Decreasing | -0.783 |

**Table S2:** Differentially abundant bacterial taxa with positive or negative magnitude of change across in the rhizospheres relative to root endosphere taxa. Data includes top 30 bacterial ASVs with a positive or negative direction of change, log fold change, and taxonomic rank.

| ASV_ID | Direction | Log fold change | Phylum | Class | Order | Family | Genus | Species |
| --- | --- | --- | --- | --- | --- | --- | --- | --- |
| ASV_2497 | Increasing | 2.161 | Acidobacteriota | Holophagae | Subgroup_7 | Subgroup_7 | Subgroup_7 | Unclassified |
| ASV_1181 | Increasing | 1.713 | Acidobacteriota | Acidobacteriae | Subgroup_2 | Subgroup_2 | Subgroup_2 | uncultured_Acidobacteria |
| ASV_3182 | Increasing | 1.706 | Acidobacteriota | Acidobacteriae | Solibacterales | Solibacteraceae | Candidatus_Solibacter | Unclassified |
| ASV_3081 | Increasing | 1.654 | Myxococcota | bacteriap25 | bacteriap25 | bacteriap25 | bacteriap25 | Unclassified |
| ASV_1085 | Increasing | 1.628 | Acidobacteriota | Acidobacteriae | Acidobacteriales | uncultured | uncultured | Unclassified |
| ASV_8300 | Increasing | 1.619 | Proteobacteria | Alphaproteobacteria | Rhizobiales | Xanthobacteraceae | uncultured | Unclassified |
| ASV_7131 | Increasing | 1.599 | Actinobacteriota | Acidimicrobiia | Microtrichales | uncultured | uncultured | uncultured_bacterium |
| ASV_3741 | Increasing | 1.592 | Proteobacteria | Alphaproteobacteria | Rhodospirillales | Magnetospiraceae | uncultured | metagenome |
| ASV_2695 | Increasing | 1.501 | Chloroflexi | Chloroflexia | Thermomicrobiales | JG30-KF-CM45 | JG30-KF-CM45 | uncultured_bacterium |
| ASV_1814 | Increasing | 1.466 | Chloroflexi | TK10 | TK10 | TK10 | TK10 | uncultured_bacterium |
| ASV_6991 | Increasing | 1.462 | Chloroflexi | Chloroflexia | Kallotenuales | AKIW781 | AKIW781 | uncultured_soil |
| ASV_2961 | Increasing | 1.443 | Verrucomicrobiota | Verrucomicrobiae | Chthoniobacterales | Chthoniobacteraceae | Candidatus_Udaeobacter | uncultured_Spartobacteria |
| ASV_317 | Increasing | 1.433 | Acidobacteriota | Acidobacteriae | Acidobacteriales | uncultured | uncultured | Unclassified |
| ASV_1480 | Increasing | 1.39 | Proteobacteria | Alphaproteobacteria | Elsterales | uncultured | uncultured | uncultured_bacterium |
| ASV_1102 | Increasing | 1.372 | Acidobacteriota | Blastocatellia | Blastocatellales | Blastocatellaceae | uncultured | uncultured_Acidobacteria |
| ASV_464 | Increasing | 1.371 | Proteobacteria | Alphaproteobacteria | Elsterales | uncultured | uncultured | uncultured_Alphaproteobacteria |
| ASV_1670 | Increasing | 1.348 | Proteobacteria | Gammaproteobacteria | Burkholderiales | Nitrosomonadaceae | MND1 | Unclassified |
| ASV_2122 | Increasing | 1.322 | Chloroflexi | KD4-96 | KD4-96 | KD4-96 | KD4-96 | Unclassified |
| ASV_1754 | Increasing | 1.315 | Gemmatimonadota | Gemmatimonadetes | Gemmatimonadales | Gemmatimonadaceae | uncultured | uncultured_Gemmatimonadales |
| ASV_8348 | Increasing | 1.307 | Acidobacteriota | Acidobacteriae | Acidobacteriales | Acidobacteriaceae_(Subgroup_1) | uncultured | Unclassified |
| ASV_331 | Increasing | 1.285 | Verrucomicrobiota | Verrucomicrobiae | Chthoniobacterales | Chthoniobacteraceae | Candidatus_Udaeobacter | uncultured_Spartobacteria |
| ASV_1661 | Increasing | 1.282 | Acidobacteriota | Blastocatellia | Blastocatellales | Blastocatellaceae | JGI_0001001-H03 | Unclassified |
| ASV_2171 | Increasing | 1.266 | Acidobacteriota | Vicinamibacteria | Vicinamibacterales | uncultured | uncultured | Unclassified |
| ASV_353 | Increasing | 1.241 | Acidobacteriota | Acidobacteriae | Acidobacteriales | uncultured | uncultured | Unclassified |
| ASV_1695 | Increasing | 1.219 | Acidobacteriota | Vicinamibacteria | Vicinamibacterales | uncultured | uncultured | Unclassified |
| ASV_579 | Increasing | 1.173 | Actinobacteriota | Thermoleophilia | Gaiellales | uncultured | uncultured | Unclassified |
| ASV_2518 | Increasing | 1.168 | Verrucomicrobiota | Verrucomicrobiae | Chthoniobacterales | Chthoniobacteraceae | Candidatus_Udaeobacter | Unclassified |
| ASV_1244 | Increasing | 1.158 | Verrucomicrobiota | Verrucomicrobiae | Chthoniobacterales | Xiphinematobacteraceae | Candidatus_Xiphinematobacter | Unclassified |
| ASV_959 | Increasing | 1.139 | Verrucomicrobiota | Verrucomicrobiae | Chthoniobacterales | Chthoniobacteraceae | Candidatus_Udaeobacter | Unclassified |
| ASV_745 | Increasing | 1.13 | Actinobacteriota | Thermoleophilia | Gaiellales | uncultured | uncultured | Unclassified |
| ASV_117 | Decreasing | -1.897 | Proteobacteria | Alphaproteobacteria | Rhizobiales | Rhodomicrobiaceae | Rhodomicrobium | uncultured_bacterium |
| ASV_54 | Decreasing | -1.955 | Actinobacteriota | Actinobacteria | Streptomycetales | Streptomycetaceae | Streptomyces | Unclassified |
| ASV_167 | Decreasing | -2 | Actinobacteriota | Actinobacteria | Streptosporangiales | Thermomonosporaceae | Actinocorallia | metagenome |
| ASV_28 | Decreasing | -2.026 | Actinobacteriota | Actinobacteria | Micromonosporales | Micromonosporaceae | Unclassified | Unclassified |
| ASV_42 | Decreasing | -2.028 | Actinobacteriota | Actinobacteria | Frankiales | Acidothermaceae | Acidothermus | Unclassified |
| ASV_73 | Decreasing | -2.051 | Actinobacteriota | Actinobacteria | Micromonosporales | Micromonosporaceae | Actinoplanes | uncultured_bacterium |
| ASV_183 | Decreasing | -2.074 | Chloroflexi | Ktedonobacteria | Ktedonobacterales | Ktedonobacteraceae | Thermosporothrix | uncultured_bacterium |
| ASV_144 | Decreasing | -2.102 | Actinobacteriota | Actinobacteria | Frankiales | Acidothermaceae | Acidothermus | uncultured_bacterium |
| ASV_264 | Decreasing | -2.107 | Proteobacteria | Gammaproteobacteria | Burkholderiales | Comamonadaceae | uncultured | Leptothrix_sp. |
| ASV_100 | Decreasing | -2.127 | Actinobacteriota | Actinobacteria | Micromonosporales | Micromonosporaceae | Unclassified | Unclassified |
| ASV_12 | Decreasing | -2.171 | Actinobacteriota | Actinobacteria | Pseudonocardiales | Pseudonocardiaceae | Pseudonocardia | Unclassified |
| ASV_49 | Decreasing | -2.185 | Actinobacteriota | Actinobacteria | Micromonosporales | Micromonosporaceae | Unclassified | Unclassified |
| ASV_17 | Decreasing | -2.226 | Actinobacteriota | Actinobacteria | Micromonosporales | Micromonosporaceae | Actinoplanes | uncultured_bacterium |
| ASV_45 | Decreasing | -2.246 | Actinobacteriota | Actinobacteria | Pseudonocardiales | Pseudonocardiaceae | Longimycelium | uncultured_bacterium |
| ASV_35 | Decreasing | -2.279 | Actinobacteriota | Actinobacteria | Pseudonocardiales | Pseudonocardiaceae | Pseudonocardia | Unclassified |
| ASV_225 | Decreasing | -2.281 | Proteobacteria | Alphaproteobacteria | Rhizobiales | Rhodomicrobiaceae | Rhodomicrobium | uncultured_bacterium |
| ASV_173 | Decreasing | -2.334 | Actinobacteriota | Actinobacteria | Frankiales | Acidothermaceae | Acidothermus | Unclassified |
| ASV_26 | Decreasing | -2.372 | Actinobacteriota | Actinobacteria | Pseudonocardiales | Pseudonocardiaceae | Actinophytocola | Actinophytocola_sp. |
| ASV_88 | Decreasing | -2.465 | Actinobacteriota | Actinobacteria | Micromonosporales | Micromonosporaceae | uncultured | uncultured_actinomycete |
| ASV_129 | Decreasing | -2.475 | Actinobacteriota | Actinobacteria | Micromonosporales | Micromonosporaceae | Virgisporangium | Unclassified |
| ASV_13 | Decreasing | -2.556 | Actinobacteriota | Actinobacteria | Pseudonocardiales | Pseudonocardiaceae | Actinophytocola | Unclassified |
| ASV_210 | Decreasing | -2.635 | Actinobacteriota | Actinobacteria | Micromonosporales | Micromonosporaceae | Actinoplanes | Unclassified |
| ASV_53 | Decreasing | -2.648 | Actinobacteriota | Actinobacteria | Pseudonocardiales | Pseudonocardiaceae | Lechevalieria | Unclassified |
| ASV_37 | Decreasing | -2.854 | Actinobacteriota | Actinobacteria | Pseudonocardiales | Pseudonocardiaceae | Actinophytocola | Unclassified |
| ASV_762 | Decreasing | -2.97 | Proteobacteria | Alphaproteobacteria | Caulobacterales | Caulobacteraceae | Asticcacaulis | uncultured_bacterium |
| ASV_81 | Decreasing | -3.179 | Actinobacteriota | Actinobacteria | Micromonosporales | Micromonosporaceae | Actinoplanes | Unclassified |
| ASV_80 | Decreasing | -3.775 | Actinobacteriota | Actinobacteria | Micromonosporales | Micromonosporaceae | Unclassified | Unclassified |
| ASV_14 | Decreasing | -4.071 | Actinobacteriota | Actinobacteria | Micromonosporales | Micromonosporaceae | Actinoplanes | uncultured_bacterium |
| ASV_3 | Decreasing | -4.672 | Proteobacteria | Alphaproteobacteria | Rickettsiales | Mitochondria | Mitochondria | Triticum_aestivum |
| ASV_1 | Decreasing | -5.196 | Proteobacteria | Alphaproteobacteria | Rickettsiales | Mitochondria | Mitochondria | Triticum_aestivum |

**Table S3:** List of endosphere HUB taxa from bacterial ASV network analysis showing taxonomic for all taxa and node degree with negative edges only.

| ASV_ID | Phylum | Class | Order | Family | Genus | Node degrees (negative edges) |
| --- | --- | --- | --- | --- | --- | --- |
| ASV_23 | Proteobacteria | Alphaproteobacteria | Rhizobiales | Xanthobacteraceae | Bradyrhizobium | 26 |
| ASV_7 | Proteobacteria | Alphaproteobacteria | Rhizobiales | Xanthobacteraceae | Unclassified | 22 |
| ASV_29 | Actinobacteriota | Actinobacteria | Frankiales | Acidothermaceae | Acidothermus | 11 |
| ASV_47 | Proteobacteria | Gammaproteobacteria | Gammaproteobacteria_Incertae_Sedis | Unknown_Family | Acidibacter | 10 |
| ASV_20 | Actinobacteriota | Actinobacteria | Pseudonocardiales | Pseudonocardiaceae | Kibdelosporangium | 9 |
| ASV_36 | Proteobacteria | Alphaproteobacteria | Elsterales | uncultured | uncultured | 8 |
| ASV_12 | Actinobacteriota | Actinobacteria | Pseudonocardiales | Pseudonocardiaceae | Pseudonocardia | 7 |
| ASV_13 | Actinobacteriota | Actinobacteria | Pseudonocardiales | Pseudonocardiaceae | Actinophytocola | 6 |
| ASV_35 | Actinobacteriota | Actinobacteria | Pseudonocardiales | Pseudonocardiaceae | Pseudonocardia | 6 |
| ASV_77 | Actinobacteriota | Actinobacteria | Pseudonocardiales | Pseudonocardiaceae | Saccharothrix | 5 |
| ASV_10 | Actinobacteriota | Actinobacteria | Streptomycetales | Streptomycetaceae | Streptomyces | 4 |
| ASV_107 | Actinobacteriota | Actinobacteria | Corynebacteriales | Mycobacteriaceae | Mycobacterium | 4 |
| ASV_119 | Proteobacteria | Alphaproteobacteria | Sphingomonadales | Sphingomonadaceae | Sphingomonas | 4 |
| ASV_19 | Actinobacteriota | Actinobacteria | Micrococcales | Promicromonosporaceae | Promicromonospora | 4 |
| ASV_28 | Actinobacteriota | Actinobacteria | Micromonosporales | Micromonosporaceae | Unclassified | 4 |
| ASV_43 | Proteobacteria | Gammaproteobacteria | Burkholderiales | Comamonadaceae | uncultured | 4 |
| ASV_117 | Proteobacteria | Alphaproteobacteria | Rhizobiales | Rhodomicrobiaceae | Rhodomicrobium | 3 |
| ASV_18 | Actinobacteriota | Actinobacteria | Pseudonocardiales | Pseudonocardiaceae | Kutzneria | 3 |
| ASV_197 | Proteobacteria | Gammaproteobacteria | Gammaproteobacteria_Incertae_Sedis | Unknown_Family | Acidibacter | 3 |
| ASV_207 | Acidobacteriota | Vicinamibacteria | Vicinamibacterales | Vicinamibacteraceae | Vicinamibacter | 3 |
| ASV_26 | Actinobacteriota | Actinobacteria | Pseudonocardiales | Pseudonocardiaceae | Actinophytocola | 3 |
| ASV_152 | Acidobacteriota | Vicinamibacteria | Vicinamibacterales | Vicinamibacteraceae | uncultured | 2 |
| ASV_154 | Acidobacteriota | Acidobacteriae | Subgroup_2 | Subgroup_2 | Subgroup_2 | 2 |
| ASV_239 | Acidobacteriota | Vicinamibacteria | Vicinamibacterales | Vicinamibacteraceae | Vicinamibacter | 2 |
| ASV_24 | Actinobacteriota | Actinobacteria | Streptomycetales | Streptomycetaceae | Streptomyces | 2 |
| ASV_306 | Proteobacteria | Gammaproteobacteria | Gammaproteobacteria_Incertae_Sedis | Unknown_Family | Acidibacter | 2 |
| ASV_313 | Proteobacteria | Alphaproteobacteria | Reyranellales | Reyranellaceae | Reyranella | 2 |
| ASV_359 | Proteobacteria | Alphaproteobacteria | Rhizobiales | Beijerinckiaceae | Microvirga | 2 |
| ASV_90 | Actinobacteriota | Actinobacteria | Pseudonocardiales | Pseudonocardiaceae | Pseudonocardia | 2 |
| ASV_1 | Proteobacteria | Alphaproteobacteria | Rickettsiales | Mitochondria | Mitochondria | 1 |
| ASV_109 | Actinobacteriota | Rubrobacteria | Rubrobacterales | Rubrobacteriaceae | Rubrobacter | 1 |
| ASV_137 | Actinobacteriota | Actinobacteria | Pseudonocardiales | Pseudonocardiaceae | Pseudonocardia | 1 |
| ASV_150 | Proteobacteria | Alphaproteobacteria | Sphingomonadales | Sphingomonadaceae | Sphingomonas | 1 |
| ASV_178 | Proteobacteria | Alphaproteobacteria | Dongiales | Dongiaceae | Dongia | 1 |
| ASV_191 | Proteobacteria | Alphaproteobacteria | Rhizobiales | Devosiaceae | Devosia | 1 |
| ASV_3 | Proteobacteria | Alphaproteobacteria | Rickettsiales | Mitochondria | Mitochondria | 1 |
| ASV_304 | Actinobacteriota | Thermoleophilia | Solirubrobacterales | 67-14 | 67-14 | 1 |
| ASV_325 | Proteobacteria | Alphaproteobacteria | Acetobacterales | Acetobacteraceae | Roseomonas | 1 |
| ASV_336 | Bacteroidota | Bacteroidia | Cytophagales | Microscillaceae | uncultured | 1 |
| ASV_518 | Proteobacteria | Alphaproteobacteria | Rhizobiales | Rhodomicrobiaceae | Rhodomicrobium | 1 |
| ASV_57 | Actinobacteriota | Actinobacteria | Frankiales | Acidothermaceae | Acidothermus | 1 |
| ASV_721 | Proteobacteria | Alphaproteobacteria | Rhizobiales | Xanthobacteraceae | uncultured | 1 |

**Table S4:** List of endosphere HUB taxa from bacterial ASV network analysis showing taxonomic for all taxa and node degree with positive edges only.

| ASV_ID | Phylum | Class | Order | Family | Genus | Node degrees (positive edges) |
| --- | --- | --- | --- | --- | --- | --- |
| ASV_13 | Actinobacteriota | Actinobacteria | Pseudonocardiales | Pseudonocardiaceae | Actinophytocola | 29 |
| ASV_12 | Actinobacteriota | Actinobacteria | Pseudonocardiales | Pseudonocardiaceae | Pseudonocardia | 25 |
| ASV_28 | Actinobacteriota | Actinobacteria | Micromonosporales | Micromonosporaceae | Unclassified | 25 |
| ASV_35 | Actinobacteriota | Actinobacteria | Pseudonocardiales | Pseudonocardiaceae | Pseudonocardia | 25 |
| ASV_19 | Actinobacteriota | Actinobacteria | Micrococcales | Promicromonosporaceae | Promicromonospora | 24 |
| ASV_77 | Actinobacteriota | Actinobacteria | Pseudonocardiales | Pseudonocardiaceae | Saccharothrix | 22 |
| ASV_26 | Actinobacteriota | Actinobacteria | Pseudonocardiales | Pseudonocardiaceae | Actinophytocola | 21 |
| ASV_117 | Proteobacteria | Alphaproteobacteria | Rhizobiales | Rhodomicrobiaceae | Rhodomicrobium | 19 |
| ASV_107 | Actinobacteriota | Actinobacteria | Corynebacteriales | Mycobacteriaceae | Mycobacterium | 16 |
| ASV_152 | Acidobacteriota | Vicinamibacteria | Vicinamibacterales | Vicinamibacteraceae | uncultured | 16 |
| ASV_197 | Proteobacteria | Gammaproteobacteria | Gammaproteobacteria_Incertae_Sedis | Unknown_Family | Acidibacter | 16 |
| ASV_119 | Proteobacteria | Alphaproteobacteria | Sphingomonadales | Sphingomonadaceae | Sphingomonas | 14 |
| ASV_20 | Actinobacteriota | Actinobacteria | Pseudonocardiales | Pseudonocardiaceae | Kibdelosporangium | 14 |
| ASV_207 | Acidobacteriota | Vicinamibacteria | Vicinamibacterales | Vicinamibacteraceae | Vicinamibacter | 14 |
| ASV_313 | Proteobacteria | Alphaproteobacteria | Reyranellales | Reyranellaceae | Reyranella | 14 |
| ASV_10 | Actinobacteriota | Actinobacteria | Streptomycetales | Streptomycetaceae | Streptomyces | 13 |
| ASV_239 | Acidobacteriota | Vicinamibacteria | Vicinamibacterales | Vicinamibacteraceae | Vicinamibacter | 13 |
| ASV_29 | Actinobacteriota | Actinobacteria | Frankiales | Acidothermaceae | Acidothermus | 13 |
| ASV_23 | Proteobacteria | Alphaproteobacteria | Rhizobiales | Xanthobacteraceae | Bradyrhizobium | 9 |
| ASV_359 | Proteobacteria | Alphaproteobacteria | Rhizobiales | Beijerinckiaceae | Microvirga | 9 |
| ASV_155 | Proteobacteria | Alphaproteobacteria | Rhizobiales | Rhizobiaceae | Allorhizobium-Neorhizobium-Pararhizobium-Rhizobium | 8 |
| ASV_74 | Actinobacteriota | Actinobacteria | Corynebacteriales | Mycobacteriaceae | Mycobacterium | 8 |
| ASV_178 | Proteobacteria | Alphaproteobacteria | Dongiales | Dongiaceae | Dongia | 7 |
| ASV_90 | Actinobacteriota | Actinobacteria | Pseudonocardiales | Pseudonocardiaceae | Pseudonocardia | 7 |
| ASV_325 | Proteobacteria | Alphaproteobacteria | Acetobacterales | Acetobacteraceae | Roseomonas | 6 |
| ASV_336 | Bacteroidota | Bacteroidia | Cytophagales | Microscillaceae | uncultured | 6 |
| ASV_357 | Myxococcota | Polyangia | Polyangiales | BIrii41 | BIrii41 | 6 |
| ASV_36 | Proteobacteria | Alphaproteobacteria | Elsterales | uncultured | uncultured | 6 |
| ASV_7 | Proteobacteria | Alphaproteobacteria | Rhizobiales | Xanthobacteraceae | Unclassified | 6 |
| ASV_129 | Actinobacteriota | Actinobacteria | Micromonosporales | Micromonosporaceae | Virgisporangium | 5 |
| ASV_154 | Acidobacteriota | Acidobacteriae | Subgroup_2 | Subgroup_2 | Subgroup_2 | 4 |
| ASV_47 | Proteobacteria | Gammaproteobacteria | Gammaproteobacteria_Incertae_Sedis | Unknown_Family | Acidibacter | 4 |
| ASV_502 | Proteobacteria | Alphaproteobacteria | Rhizobiales | Rhizobiaceae | Unclassified | 4 |
| ASV_54 | Actinobacteriota | Actinobacteria | Streptomycetales | Streptomycetaceae | Streptomyces | 4 |
| ASV_57 | Actinobacteriota | Actinobacteria | Frankiales | Acidothermaceae | Acidothermus | 4 |
| ASV_84 | Actinobacteriota | Actinobacteria | Propionibacteriales | Nocardioidaceae | Kribbella | 4 |
| ASV_18 | Actinobacteriota | Actinobacteria | Pseudonocardiales | Pseudonocardiaceae | Kutzneria | 3 |
| ASV_43 | Proteobacteria | Gammaproteobacteria | Burkholderiales | Comamonadaceae | uncultured | 3 |
| ASV_518 | Proteobacteria | Alphaproteobacteria | Rhizobiales | Rhodomicrobiaceae | Rhodomicrobium | 3 |
| ASV_520 | Bacteroidota | Bacteroidia | Cytophagales | Microscillaceae | Unclassified | 3 |
| ASV_53 | Actinobacteriota | Actinobacteria | Pseudonocardiales | Pseudonocardiaceae | Lechevalieria | 3 |
| ASV_88 | Actinobacteriota | Actinobacteria | Micromonosporales | Micromonosporaceae | uncultured | 3 |
| ASV_137 | Actinobacteriota | Actinobacteria | Pseudonocardiales | Pseudonocardiaceae | Pseudonocardia | 2 |
| ASV_150 | Proteobacteria | Alphaproteobacteria | Sphingomonadales | Sphingomonadaceae | Sphingomonas | 2 |
| ASV_160 | Proteobacteria | Alphaproteobacteria | Rhizobiales | Rhizobiaceae | Unclassified | 2 |
| ASV_180 | Proteobacteria | Alphaproteobacteria | Rhizobiales | Rhizobiaceae | Phyllobacterium | 2 |
| ASV_191 | Proteobacteria | Alphaproteobacteria | Rhizobiales | Devosiaceae | Devosia | 2 |
| ASV_285 | Proteobacteria | Gammaproteobacteria | Steroidobacterales | Steroidobacteraceae | Steroidobacter | 2 |
| ASV_304 | Actinobacteriota | Thermoleophilia | Solirubrobacterales | 67-14 | 67-14 | 2 |
| ASV_462 | Proteobacteria | Alphaproteobacteria | Micropepsales | Micropepsaceae | uncultured | 2 |
| ASV_67 | Actinobacteriota | Actinobacteria | Pseudonocardiales | Pseudonocardiaceae | Lechevalieria | 2 |
| ASV_721 | Proteobacteria | Alphaproteobacteria | Rhizobiales | Xanthobacteraceae | uncultured | 2 |
| ASV_1 | Proteobacteria | Alphaproteobacteria | Rickettsiales | Mitochondria | Mitochondria | 1 |
| ASV_103 | Proteobacteria | Alphaproteobacteria | Rhizobiales | Beijerinckiaceae | Unclassified | 1 |
| ASV_109 | Actinobacteriota | Rubrobacteria | Rubrobacterales | Rubrobacteriaceae | Rubrobacter | 1 |
| ASV_112 | Actinobacteriota | Actinobacteria | Corynebacteriales | Mycobacteriaceae | Mycobacterium | 1 |
| ASV_115 | Proteobacteria | Alphaproteobacteria | Rickettsiales | Mitochondria | Mitochondria | 1 |
| ASV_122 | Actinobacteriota | Actinobacteria | Micromonosporales | Micromonosporaceae | Unclassified | 1 |
| ASV_130 | Actinobacteriota | Actinobacteria | Pseudonocardiales | Pseudonocardiaceae | Pseudonocardia | 1 |
| ASV_138 | Acidobacteriota | Acidobacteriae | Bryobacterales | Bryobacteraceae | Bryobacter | 1 |
| ASV_15 | Actinobacteriota | Actinobacteria | Streptomycetales | Streptomycetaceae | Streptomyces | 1 |
| ASV_198 | Proteobacteria | Alphaproteobacteria | Rhizobiales | Rhizobiaceae | Mesorhizobium | 1 |
| ASV_218 | Actinobacteriota | Thermoleophilia | Solirubrobacterales | Solirubrobacteraceae | Conexibacter | 1 |
| ASV_240 | Chloroflexi | Chloroflexia | Thermomicrobiales | JG30-KF-CM45 | JG30-KF-CM45 | 1 |
| ASV_242 | Acidobacteriota | Acidobacteriae | Solibacterales | Solibacteraceae | Candidatus_Solibacter | 1 |
| ASV_281 | Actinobacteriota | Actinobacteria | Micrococcales | Microbacteriaceae | Agromyces | 1 |
| ASV_3 | Proteobacteria | Alphaproteobacteria | Rickettsiales | Mitochondria | Mitochondria | 1 |
| ASV_306 | Proteobacteria | Gammaproteobacteria | Gammaproteobacteria_Incertae_Sedis | Unknown_Family | Acidibacter | 1 |
| ASV_33 | Proteobacteria | Alphaproteobacteria | Rickettsiales | Mitochondria | Mitochondria | 1 |
| ASV_34 | Actinobacteriota | Actinobacteria | Streptomycetales | Streptomycetaceae | Streptomyces | 1 |
| ASV_37 | Actinobacteriota | Actinobacteria | Pseudonocardiales | Pseudonocardiaceae | Actinophytocola | 1 |
| ASV_380 | Acidobacteriota | Acidobacteriae | Acidobacteriales | Acidobacteriaceae_(Subgroup_1) | Occallatibacter | 1 |
| ASV_388 | Actinobacteriota | Actinobacteria | Propionibacteriales | Nocardioidaceae | Nocardioides | 1 |
| ASV_395 | Proteobacteria | Alphaproteobacteria | Dongiales | Dongiaceae | Dongia | 1 |
| ASV_42 | Actinobacteriota | Actinobacteria | Frankiales | Acidothermaceae | Acidothermus | 1 |
| ASV_49 | Actinobacteriota | Actinobacteria | Micromonosporales | Micromonosporaceae | Unclassified | 1 |
| ASV_62 | Actinobacteriota | Actinobacteria | Frankiales | Geodermatophilaceae | Geodermatophilus | 1 |
| ASV_79 | Proteobacteria | Alphaproteobacteria | Sphingomonadales | Sphingomonadaceae | Sphingomonas | 1 |
| ASV_8 | Actinobacteriota | Actinobacteria | Pseudonocardiales | Pseudonocardiaceae | Amycolatopsis | 1 |
| ASV_80 | Actinobacteriota | Actinobacteria | Micromonosporales | Micromonosporaceae | Unclassified | 1 |

**Table S5:** List of rhizosphere HUB taxa from bacterial ASV network analysis showing taxonomic for all taxa and node degree with negative edges only.

| ASV_ID | Phylum | Class | Order | Family | Genus | Node degrees (negative edges) |
| --- | --- | --- | --- | --- | --- | --- |
| ASV_7 | Proteobacteria | Alphaproteobacteria | Rhizobiales | Xanthobacteraceae | Unclassified | 20 |
| ASV_23 | Proteobacteria | Alphaproteobacteria | Rhizobiales | Xanthobacteraceae | Bradyrhizobium | 11 |
| ASV_109 | Actinobacteriota | Rubrobacteria | Rubrobacterales | Rubrobacteriaceae | Rubrobacter | 4 |
| ASV_36 | Proteobacteria | Alphaproteobacteria | Elsterales | uncultured | uncultured | 4 |
| ASV_240 | Chloroflexi | Chloroflexia | Thermomicrobiales | JG30-KF-CM45 | JG30-KF-CM45 | 3 |
| ASV_10 | Actinobacteriota | Actinobacteria | Streptomycetales | Streptomycetaceae | Streptomyces | 2 |
| ASV_12245 | Acidobacteriota | Vicinamibacteria | Vicinamibacterales | Vicinamibacteraceae | Vicinamibacteraceae | 2 |
| ASV_185 | Actinobacteriota | Rubrobacteria | Rubrobacterales | Rubrobacteriaceae | Rubrobacter | 2 |
| ASV_317 | Acidobacteriota | Acidobacteriae | Acidobacteriales | uncultured | uncultured | 2 |
| ASV_490 | Actinobacteriota | Rubrobacteria | Rubrobacterales | Rubrobacteriaceae | Rubrobacter | 2 |
| ASV_497 | Actinobacteriota | Rubrobacteria | Rubrobacterales | Rubrobacteriaceae | Rubrobacter | 2 |
| ASV_683 | Proteobacteria | Alphaproteobacteria | Rhizobiales | Beijerinckiaceae | Microvirga | 2 |
| ASV_79 | Proteobacteria | Alphaproteobacteria | Sphingomonadales | Sphingomonadaceae | Sphingomonas | 2 |
| ASV_950 | Actinobacteriota | Rubrobacteria | Rubrobacterales | Rubrobacteriaceae | Rubrobacter | 2 |
| ASV_107 | Actinobacteriota | Actinobacteria | Corynebacteriales | Mycobacteriaceae | Mycobacterium | 1 |
| ASV_119 | Proteobacteria | Alphaproteobacteria | Sphingomonadales | Sphingomonadaceae | Sphingomonas | 1 |
| ASV_12 | Actinobacteriota | Actinobacteria | Pseudonocardiales | Pseudonocardiaceae | Pseudonocardia | 1 |
| ASV_152 | Acidobacteriota | Vicinamibacteria | Vicinamibacterales | Vicinamibacteraceae | uncultured | 1 |
| ASV_1670 | Proteobacteria | Gammaproteobacteria | Burkholderiales | Nitrosomonadaceae | MND1 | 1 |
| ASV_2034 | Actinobacteriota | Acidimicrobiia | Microtrichales | Iamiaceae | Iamia | 1 |
| ASV_239 | Acidobacteriota | Vicinamibacteria | Vicinamibacterales | Vicinamibacteraceae | Vicinamibacter | 1 |
| ASV_259 | Proteobacteria | Gammaproteobacteria | Xanthomonadales | Xanthomonadaceae | Luteimonas | 1 |
| ASV_262 | Chloroflexi | Chloroflexia | Thermomicrobiales | JG30-KF-CM45 | JG30-KF-CM45 | 1 |
| ASV_2718 | Proteobacteria | Gammaproteobacteria | PLTA13 | PLTA13 | PLTA13 | 1 |
| ASV_313 | Proteobacteria | Alphaproteobacteria | Reyranellales | Reyranellaceae | Reyranella | 1 |
| ASV_402 | Actinobacteriota | Thermoleophilia | Gaiellales | Unclassified | Unclassified | 1 |
| ASV_407 | Actinobacteriota | Rubrobacteria | Rubrobacterales | Rubrobacteriaceae | Rubrobacter | 1 |
| ASV_488 | Proteobacteria | Alphaproteobacteria | Rhizobiales | Beijerinckiaceae | Microvirga | 1 |
| ASV_6 | Actinobacteriota | Actinobacteria | Streptomycetales | Streptomycetaceae | Streptomyces | 1 |
| ASV_655 | Actinobacteriota | Rubrobacteria | Rubrobacterales | Rubrobacteriaceae | Rubrobacter | 1 |
| ASV_674 | Actinobacteriota | Actinobacteria | Propionibacteriales | Propionibacteriaceae | Microlunatus | 1 |
| ASV_704 | Actinobacteriota | Thermoleophilia | Solirubrobacterales | 67-14 | 67-14 | 1 |
| ASV_816 | Acidobacteriota | Acidobacteriae | Acidobacteriales | Acidobacteriaceae_(Subgroup_1) | uncultured | 1 |
| ASV_987 | Chloroflexi | Chloroflexia | Thermomicrobiales | JG30-KF-CM45 | JG30-KF-CM45 | 1 |

**Table S6**: List of rhizosphere HUB taxa from bacterial ASV network analysis showing taxonomic for all taxa and node degree with positive edges only.

| ASV_ID | Phylum | Class | Order | Family | Genus | Node degrees (positive edges) |
| --- | --- | --- | --- | --- | --- | --- |
| ASV_109 | Actinobacteriota | Rubrobacteria | Rubrobacterales | Rubrobacteriaceae | Rubrobacter | 18 |
| ASV_497 | Actinobacteriota | Rubrobacteria | Rubrobacterales | Rubrobacteriaceae | Rubrobacter | 12 |
| ASV_185 | Actinobacteriota | Rubrobacteria | Rubrobacterales | Rubrobacteriaceae | Rubrobacter | 11 |
| ASV_240 | Chloroflexi | Chloroflexia | Thermomicrobiales | JG30-KF-CM45 | JG30-KF-CM45 | 9 |
| ASV_683 | Proteobacteria | Alphaproteobacteria | Rhizobiales | Beijerinckiaceae | Microvirga | 9 |
| ASV_10 | Actinobacteriota | Actinobacteria | Streptomycetales | Streptomycetaceae | Streptomyces | 8 |
| ASV_262 | Chloroflexi | Chloroflexia | Thermomicrobiales | JG30-KF-CM45 | JG30-KF-CM45 | 8 |
| ASV_987 | Chloroflexi | Chloroflexia | Thermomicrobiales | JG30-KF-CM45 | JG30-KF-CM45 | 8 |
| ASV_674 | Actinobacteriota | Actinobacteria | Propionibacteriales | Propionibacteriaceae | Microlunatus | 6 |
| ASV_152 | Acidobacteriota | Vicinamibacteria | Vicinamibacterales | Vicinamibacteraceae | uncultured | 5 |
| ASV_313 | Proteobacteria | Alphaproteobacteria | Reyranellales | Reyranellaceae | Reyranella | 5 |
| ASV_389 | Actinobacteriota | Rubrobacteria | Rubrobacterales | Rubrobacteriaceae | Rubrobacter | 5 |
| ASV_407 | Actinobacteriota | Rubrobacteria | Rubrobacterales | Rubrobacteriaceae | Rubrobacter | 5 |
| ASV_488 | Proteobacteria | Alphaproteobacteria | Rhizobiales | Beijerinckiaceae | Microvirga | 5 |
| ASV_655 | Actinobacteriota | Rubrobacteria | Rubrobacterales | Rubrobacteriaceae | Rubrobacter | 5 |
| ASV_1197 | Actinobacteriota | Rubrobacteria | Rubrobacterales | Rubrobacteriaceae | Rubrobacter | 4 |
| ASV_359 | Proteobacteria | Alphaproteobacteria | Rhizobiales | Beijerinckiaceae | Microvirga | 4 |
| ASV_402 | Actinobacteriota | Thermoleophilia | Gaiellales | Unclassified | Unclassified | 4 |
| ASV_490 | Actinobacteriota | Rubrobacteria | Rubrobacterales | Rubrobacteriaceae | Rubrobacter | 4 |
| ASV_107 | Actinobacteriota | Actinobacteria | Corynebacteriales | Mycobacteriaceae | Mycobacterium | 3 |
| ASV_1102 | Acidobacteriota | Blastocatellia | Blastocatellales | Blastocatellaceae | uncultured | 3 |
| ASV_12 | Actinobacteriota | Actinobacteria | Pseudonocardiales | Pseudonocardiaceae | Pseudonocardia | 3 |
| ASV_331 | Verrucomicrobiota | Verrucomicrobiae | Chthoniobacterales | Chthoniobacteraceae | Candidatus_Udaeobacter | 3 |
| ASV_36 | Proteobacteria | Alphaproteobacteria | Elsterales | uncultured | uncultured | 3 |
| ASV_1670 | Proteobacteria | Gammaproteobacteria | Burkholderiales | Nitrosomonadaceae | MND1 | 2 |
| ASV_202 | Actinobacteriota | Thermoleophilia | Solirubrobacterales | Solirubrobacteraceae | Solirubrobacter | 2 |
| ASV_23 | Proteobacteria | Alphaproteobacteria | Rhizobiales | Xanthobacteraceae | Bradyrhizobium | 2 |
| ASV_464 | Proteobacteria | Alphaproteobacteria | Elsterales | uncultured | uncultured | 2 |
| ASV_7 | Proteobacteria | Alphaproteobacteria | Rhizobiales | Xanthobacteraceae | Unclassified | 2 |
| ASV_950 | Actinobacteriota | Rubrobacteria | Rubrobacterales | Rubrobacteriaceae | Rubrobacter | 2 |
| ASV_104 | Actinobacteriota | Actinobacteria | Streptomycetales | Streptomycetaceae | Streptomyces | 1 |
| ASV_1139 | Actinobacteriota | Thermoleophilia | Gaiellales | Gaiellaceae | Gaiella | 1 |
| ASV_1715 | Acidobacteriota | Vicinamibacteria | Vicinamibacterales | Vicinamibacteraceae | Vicinamibacteraceae | 1 |
| ASV_242 | Acidobacteriota | Acidobacteriae | Solibacterales | Solibacteraceae | Candidatus_Solibacter | 1 |
| ASV_259 | Proteobacteria | Gammaproteobacteria | Xanthomonadales | Xanthomonadaceae | Luteimonas | 1 |
| ASV_268 | Actinobacteriota | Actinobacteria | Micrococcales | Microbacteriaceae | Agromyces | 1 |
| ASV_292 | Acidobacteriota | Acidobacteriae | Acidobacteriales | Acidobacteriaceae_(Subgroup_1) | Unclassified | 1 |
| ASV_317 | Acidobacteriota | Acidobacteriae | Acidobacteriales | uncultured | uncultured | 1 |
| ASV_34 | Actinobacteriota | Actinobacteria | Streptomycetales | Streptomycetaceae | Streptomyces | 1 |
| ASV_348 | Acidobacteriota | Acidobacteriae | Acidobacteriales | Acidobacteriaceae_(Subgroup_1) | Granulicella | 1 |
| ASV_353 | Acidobacteriota | Acidobacteriae | Acidobacteriales | uncultured | uncultured | 1 |
| ASV_377 | Acidobacteriota | Acidobacteriae | Bryobacterales | Bryobacteraceae | Bryobacter | 1 |
| ASV_380 | Acidobacteriota | Acidobacteriae | Acidobacteriales | Acidobacteriaceae_(Subgroup_1) | Occallatibacter | 1 |
| ASV_579 | Actinobacteriota | Thermoleophilia | Gaiellales | uncultured | uncultured | 1 |
| ASV_587 | Proteobacteria | Alphaproteobacteria | Micropepsales | Micropepsaceae | uncultured | 1 |
| ASV_601 | Proteobacteria | Alphaproteobacteria | Rhizobiales | Beijerinckiaceae | Unclassified | 1 |
| ASV_638 | Acidobacteriota | Acidobacteriae | Acidobacteriales | uncultured | uncultured | 1 |
| ASV_704 | Actinobacteriota | Thermoleophilia | Solirubrobacterales | 67-14 | 67-14 | 1 |
| ASV_742 | Acidobacteriota | Acidobacteriae | Bryobacterales | Bryobacteraceae | Bryobacter | 1 |
| ASV_79 | Proteobacteria | Alphaproteobacteria | Sphingomonadales | Sphingomonadaceae | Sphingomonas | 1 |
| ASV_816 | Acidobacteriota | Acidobacteriae | Acidobacteriales | Acidobacteriaceae_(Subgroup_1) | uncultured | 1 |
| ASV_879 | Actinobacteriota | Thermoleophilia | Gaiellales | uncultured | uncultured | 1 |
| ASV_8814 | Acidobacteriota | Vicinamibacteria | Vicinamibacterales | Vicinamibacteraceae | Vicinamibacteraceae | 1 |
| ASV_898 | Chloroflexi | KD4-96 | KD4-96 | KD4-96 | KD4-96 | 1 |
